# Exploring the druggability of the binding site of aurovertin, an exogenous allosteric inhibitor of F_O_F_1_-ATP synthase

**DOI:** 10.3389/fphar.2022.1012008

**Published:** 2022-10-14

**Authors:** Luis Fernando Cofas-Vargas, Paola Mendoza-Espinosa, Luis Pablo Avila-Barrientos, Diego Prada-Gracia, Héctor Riveros-Rosas, Enrique García-Hernández

**Affiliations:** ^1^ Universidad Nacional Autónoma de México, Instituto de Química, Ciudad Universitaria, Mexico City, Mexico; ^2^ Tecnologico de Monterrey, The Institute for Obesity Research, Monterrey, Mexico; ^3^ Unidad de Investigación en Biología Computacional y Diseño de Fármacos, Hospital Infantil de México Federico Gómez, Mexico City, Mexico; ^4^ Departamento de Bioquímica, Facultad de Medicina, Universidad Nacional Autónoma de México, Avenida Universidad 3000, Cd. Universitaria, Mexico City, Mexico

**Keywords:** FOF1-ATP synthase inhibition, conformational dynamics, solvent sites, binding free energy, hot spot binding residues, bacterial pathogens

## Abstract

In addition to playing a central role in the mitochondria as the main producer of ATP, F_O_F_1_-ATP synthase performs diverse key regulatory functions in the cell membrane. Its malfunction has been linked to a growing number of human diseases, including hypertension, atherosclerosis, cancer, and some neurodegenerative, autoimmune, and aging diseases. Furthermore, inhibition of this enzyme jeopardizes the survival of several bacterial pathogens of public health concern. Therefore, F_O_F_1_-ATP synthase has emerged as a novel drug target both to treat human diseases and to combat antibiotic resistance. In this work, we carried out a computational characterization of the binding sites of the fungal antibiotic aurovertin in the bovine F_1_ subcomplex, which shares a large identity with the human enzyme. Molecular dynamics simulations showed that although the binding sites can be described as preformed, the inhibitor hinders inter-subunit communications and exerts long-range effects on the dynamics of the catalytic site residues. End-point binding free energy calculations revealed hot spot residues for aurovertin recognition. These residues were also relevant to stabilize solvent sites determined from mixed-solvent molecular dynamics, which mimic the interaction between aurovertin and the enzyme, and could be used as pharmacophore constraints in virtual screening campaigns. To explore the possibility of finding species-specific inhibitors targeting the aurovertin binding site, we performed free energy calculations for two bacterial enzymes with experimentally solved 3D structures. Finally, an analysis of bacterial sequences was carried out to determine conservation of the aurovertin binding site. Taken together, our results constitute a first step in paving the way for structure-based development of new allosteric drugs targeting F_O_F_1_-ATP synthase sites of exogenous inhibitors.

## 1 Introduction

Because of its crucial role in the production of ATP and its involvement in regulating multiple physiological processes in plasma membranes, an improper function of F_O_F_1_-ATP synthase may trigger various diseases in humans, including Alzheimer, Parkinson, amyotrophic lateral sclerosis, diabetes, hypertension, and cancer (Nesci et al., 2019; Galber et al., 2021). There is evidence that F_O_F_1_-ATP synthase inhibition results in arrest of both tumor angiogenesis and metastasis (Taurino and Gnoni, 2018). In addition, this enzyme is an attractive new drug target for combating the growing problem of antimicrobial resistance by undermining bacterial bioenergetics (Cook et al., 2014; Hards and Cook, 2018; Ruiz-Blanco et al., 2021; Avila-Barrientos et al., 2022). A salient achievement of the latter has been the design of bedaquiline, a drug used to treat tuberculosis (Lakshmanan and Xavier, 2013; Luo et al., 2020), which spawned the idea of using F_O_F_1_-ATP synthase as a species-specific antimicrobial target. Moreover, there is strong evidence that the inhibition of this enzyme can effectively assist in the interruption of the life cycle of facultative anaerobes with multiresistance, as has been shown for several species of the genera *Streptococcus*, *Staphylococcus*, *Escherichia,* and *Klebsiella* (Bosch et al., 2020; Vestergaard et al., 2021).

**FIGURE 1:**
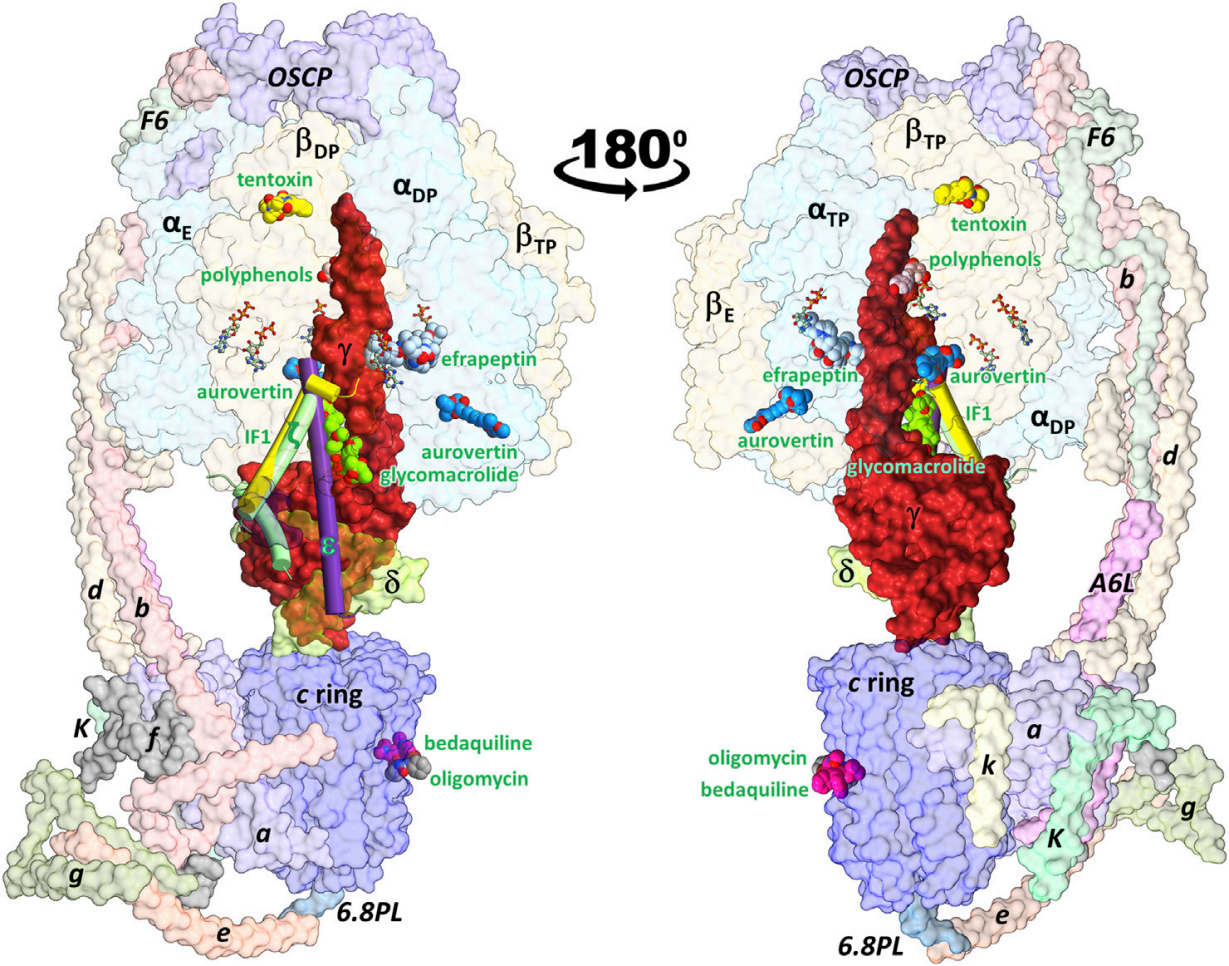
Schematic representation of the ATP synthase architecture and the binding sites of exogenous and endogenous allosteric inhibitors. Inhibitors whose 3D structure in complex with the enzyme has been solved experimentally were superimposed on the cryoEM structure of the porcine enzyme in an inhibited conformation by the IF1 subunit (PDB ID: 6j5i). The minimal structures of F_1_ and F_O_ subcomplexes are composed of the α_3_β_3_γδε and ab_2_c_8-17_ subunits, respectively. Eukaryotic ATP synthases contain supernumerary subunits, mainly in the F_O_ sector. F_O_F_1_-ATP synthase inhibitors can be classified as endogenous or exogenous. The endogenous inhibitory ε (PDB ID: 6oqr (Sobti et al., 2020)), ζ (PDB ID: 5dn6 (Morales-Rios et al., 2015)) and IF1 (PDB ID: 6j5i (Gu et al., 2019)) subunits are shown in purple, green and yellow cylinders, respectively. The exogenous inhibitors aurovertin B (PDB ID: 1cow (van Raaij et al., 1996)), resveratrol (PDB ID: 2jiz (Gledhill et al., 2007a)), tentoxin (PDB ID: 1 kmh (Groth and Pohl, 2001)), efrapeptin (PDB ID: 1efr (Abrahams et al., 1996)), oligomycin (PDB ID: 6cp5 (Srivastava et al., 2018)), and glycomacrolide apoptolidin A (PDB ID: 7MD3 (Reisman et al., 2022)) are shown in spheres. All inhibitors are labeled in green. β_TP_ and β_E_ each bind to one aurovertin molecule. Also shown is the position of the antitubercular drug bedaquiline (PDB ID: 7jg8 (Guo et al., 2021)), which occupies *c*-ring sites equivalent to those of oligomycin. Nucleotides are shown in balls-and-sticks.

All F_O_F_1_-ATP synthases share a basic architecture composed of a transmembrane F_O_ subcomplex and a solvent-exposed F_1_ subcomplex (Figure 1) (Hong and Pedersen, 2003; Kühlbrandt, 2019). F_O_ drives rotary motion of the rod-shaped γ subunit using the electrochemical gradient established by the respiratory chain. F_1_ carries the catalytic machinery comprising a hexamer of alternating pairs of α/β subunits in which the γ subunit is embedded. The ε subunit (δ in mitochondrial ATP synthases) is bound to a solvent-exposed region of the γ subunit. Each of the homologous three-domain α and β subunits contains a nucleotide binding site, but only the β subunits, with the participation of a few key residues of a neighboring α subunit, are catalytic (Pulido et al., 2010; Salcedo et al., 2014). Following the nucleotide occupancy observed in the first crystal F_1_ structure of *Bos taurus* (BtF_1_) (Abrahams et al., 1994), the β subunits are commonly referred to as β_E_ (empty catalytic site), β_DP_ (ADP bound), and β_TP_ (ATP bound), although other nucleotide occupancies and conformations have been seen in later structures (Kühlbrandt, 2019). β_E_ shows an open conformation, with the C-terminal domain (CTD) largely exposed to the solvent. β_DP_ and β_TP_ adopt closed conformations that largely overlap each other, although β_DP_ packs more extensively against its adjacent subunits. These conformations, in which each β subunit makes unique contacts with the central asymmetric γ subunit, constitute the structural basis of the binding change mechanisms that involves the alternate conformational changes of the β subunits coupled to rotation of the γ subunit (Abrahams et al., 1994).

Modern medicine requires the identification and validation of novel key therapeutic targets to tackle diseases lacking an effective cure, or whose treatment causes significant side effects. The vast majority of drugs approved for clinical use (∼99.5%) bind directly to active sites (Sheik Amamuddy et al., 2020). Side effects associated with these orthosteric binders are often related to cross-reactivity with off-target proteins. Instead, allosteric molecules bind to pockets that are less conserved than active sites (Lu et al., 2019; Bhat et al., 2020; Chatzigoulas and Cournia, 2021), providing the opportunity to more selectively modulate targets of interest (Nesci et al., 2019; Chatzigoulas and Cournia, 2021). In particular, allosteric sites that lack endogenous binders (i.e., non-functional allosteric sites) are attractive, as binders designed against them typically require lower potency optimization than drugs raised against orthosteric or allosteric functional sites (Nussinov and Tsai, 2012; Chatzigoulas and Cournia, 2021). In this context, it is relevant that biological evolution has “discovered” hundreds of exogenous molecules that bind to non-functional allosteric sites of F_O_F_1_-ATP synthase, blocking the progression of conformational changes that drive the enzyme’s rotary mechanism. This aspect could be particularly useful for the design of specific F_O_F_1_-ATP synthase inhibitors, since some regions of the active site of this enzyme are highly conserved in P-loop NTPases (Walker et al., 1982; Leipe et al., 2003).

As illustrated in Figure 1, the binding sites of several allosteric inhibitors have been structurally determined throughout the enzyme (Hong and Pedersen, 2008; Patel et al., 2020). To prevent wasteful consumption of ATP, the ε subunit in bacteria and chloroplast, and the supernumerary subunits IF1 and ζ in eukaryotes and α-proteobacteria, respectively, embed a helical domain into a transient cavity formed between α, β and γ subunits, suppressing enzyme rotation (Cingolani and Duncan, 2011; Varghese et al., 2018; Jarman et al., 2021). Similarly, the glycomacrolides apoptolidin A and ammocidin A bind to the same pocket as the endogenous inhibitory subunits (Reisman et al., 2022). Interestingly, these two exogenous binders with unknown biological function demonstrated efficacy in suppressing leukemia progression. Bacteria and fungi have developed numerous molecules to inhibit the F_O_F_1_-ATP synthases of other organisms as a means of self-defense, invasion, or to eliminate competition from other species. The herbicide tentoxin and the antibiotics efrapeptins and aurovertins bind to nonoverlapping pockets at α/β interfaces (Abrahams et al., 1996; van Raaij et al., 1996; Groth and Pohl, 2001). The antibiotic oligomycin binds to an equivalent site of bedaquiline on the c subunits in F_O_ (Symersky et al., 2012). Plant polyphenols such as resveratrol, piceatannol, and quercetin bind to a transient cavity formed between α, β and γ subunits, although the functional relevance of their inhibitory effect remains unclear (Gledhill et al., 2007a).

The intersubunit communication events that occur along the rotary mechanism of ATP synthase involve the formation and rupture of multiple pockets. As described above, nature has exploited these transient pockets as sites for allosteric inhibition, in which peptides and small molecules insert like wedges into a gear, preventing progression of the rotary cycle. Therefore, it could be hypothesized they are exploitable pockets to develop potential pharmacological allosteric modulators of this enzyme (Huang et al., 2008; Patel et al., 2020). However, there are no systematic studies aimed at exploring the druggability potential of F_O_F_1_-ATP synthase pockets to which exogenous molecules bind. As a first step to pave the way for structure-based development of new allosteric drugs targeting F_O_F_1_-ATP synthase, in this work we carried out a computational characterization of the binding sites of the fungal antibiotic aurovertin (AUR). The ability of AUR to inhibit the development of malignant cell lines or tumors through specific binding to F_O_F_1_-ATP synthase makes it an attractive model compound to treat different cancer types (Wu et al., 2020). Although not yet experimentally explored, AUR could also cause effects comparable to those elicited by other F_O_F_1_-ATP synthase inhibitors in other diseases (Wang et al., 2021), including treatment against bacterial pathogens (Mahajan, 2013). AURs are a group of fungal reduced polyketides with antitumor, nematicidal, and antimicrobial activities that contain a 2,6-dioxabicyclo [3.2.1]octane core attached to a methylated α-pyrone through a triene linker (Patel et al., 2020; Wu et al., 2020; Flores Francisco et al., 2021). There are 21 known AURs (termed A to U). The differences between them are found mainly in the bicyclo moiety, where up to four substitutions can occur, mainly by hydrophobic groups. These molecules are mixed inhibitors. They completely stop ATP synthesis and leave a significant residual activity (up to 40%) in the direction of hydrolysis. The basis for this differential effect on both catalytic activities remains unclear (van Raaij et al., 1996; Johnson et al., 2009).

**FIGURE 2:**
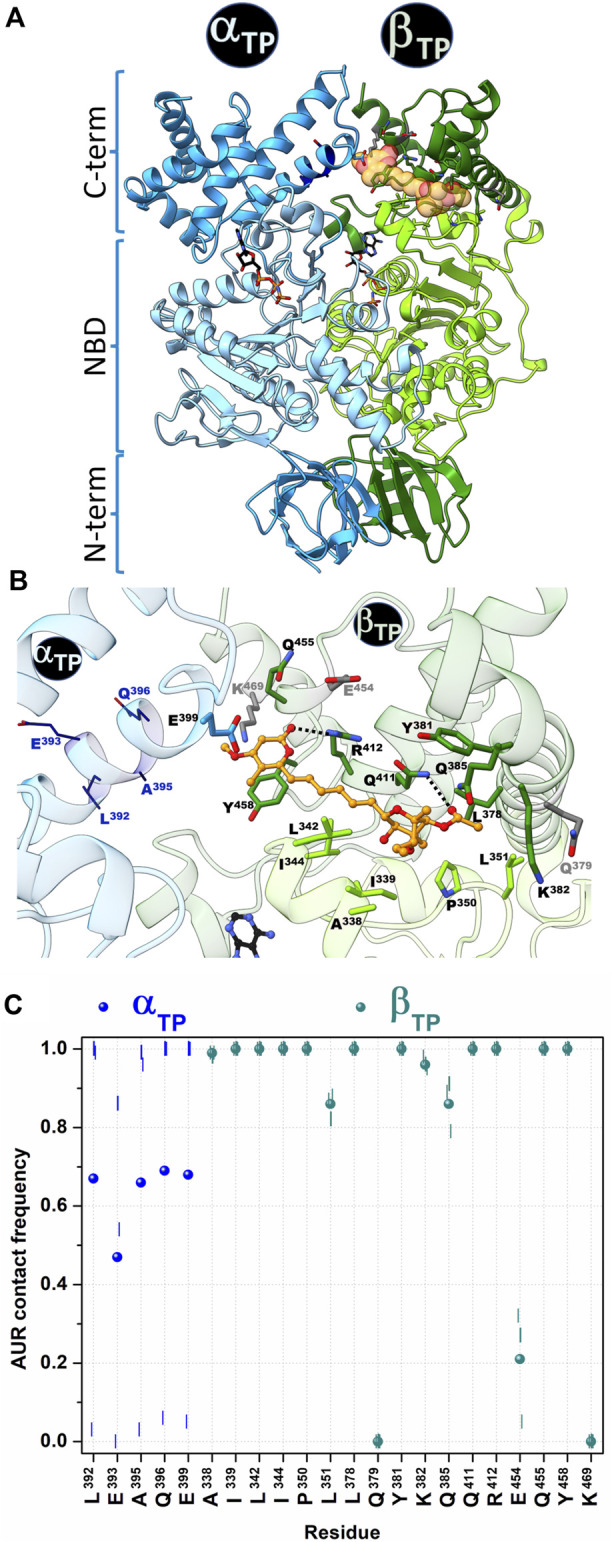
Contact analysis at the AUR binding site in β_TP_. In the crystal structure of BtF_1_ complexed with AUR B (PDB ID: 1cow), one inhibitor molecule is bound to β_TP_ and a second to β_E_. Only the β_TP_ site is shown. **(A)** Both α and β subunits have a three-domain organization: a β-barrel N-terminal domain (NTD), a central nucleotide binding domain (NBD) and a C-terminal helical domain (CTD). The aurovertin binding sites are in equivalent positions in β_E_ and β_TP_, in a cavity between NBD and CTD. AUR and nucleotides are shown in yellow spheres and sticks, respectively. **(B)** Structure of the AUR binding site. Residues in the crystal structure within 5 Å of the inhibitor are shown in sticks. Three β-subunit residues that lost contact with AUR during MD simulations are in gray. Four α-subunit residues (shown in wireframe) that were initially >5 Å from the inhibitor came into close contact with it during the simulations. AUR is shown in balls-and-sticks. The hydrogen bonds between βR^412^-O19 and βQ^411^-O25, which remained formed throughout the simulations, are shown with black doted lines **(C)** AUR-protein interaction cumulative frequency observed in the MD simulations. The results of the individual trajectories are shown as vertical lines; the circle symbols correspond to the mean values of the three replicas.

The crystal structure of the BtF_1_ in complex with aurovertin B (AUR B) shows two inhibitor binding sites, one in β_E_ and one in β_TP_, in a hydrophobic cleft between the nucleotide binding domain (NBD) and CTD (Figure 1 and Figure 2A). The closest distance between AUR B and ATP in β_TP_ is ∼12 Å, which is consistent with the observed uncompetitive inhibition (van Raaij et al., 1996). Because of a tighter packing, no space for the inhibitor is available in β_DP_. Thus, the inhibition mechanism has been proposed to consist in sterically preventing the conversion of β_TP_ to β_DP_ (hydrolysis direction) or β_E_ to β_DP_ (synthesis direction) (van Raaij et al., 1996). AUR B shows the same binding mode at the β_TP_ and β_E_ sites (β_TP_-AUR^+^ and β_E_-AUR^+^, respectively) interacting with an identical set of 17 β-subunit residues within 5 Å of the inhibitor (Figure 2B). Each site is composed of 1) six hydrophobic NBD residues (βA^338^, βI^339^, βL^342^ in the last NBD helix, and βI^344^, βP^350^, βL^351^ in the linker preceding CTD), and 2) eleven CTD residues: three hydrophobic residues (βL^378^, βY^381^ in the helix-turn-helix motif (HTH), and βY^458^ in the last loop) and eight polar residues (βQ^379^, βK^382^, βQ^385^, βQ^411^, βR^412^ in HTH, and βE^454^, βQ^455^, βK^469^ in the last two helices of the protein). The polar residues contact the inhibitor mainly with their nonpolar moieties, so the interaction is predominantly hydrophobic. The side chains of βQ^411^ and βR^412^ form a hydrogen bond with the bicyclo carbonyl (O25) and pyrone (O19) oxygens, respectively. The pyrone forms a π-π stacking with βY^458^. The β_TP_ binding site additionally contains αE^399^, residue in HTH of α_TP_, which makes a van der Waals contact with the AUR B O17 atom (>20 Å in β_E_).

In this work, we carried out a computational characterization of the AUR binding sites with the aim of shedding new insights into the structural and energetic basis of inhibitor recognition for the future development of modulators of F_O_F_1_-ATP synthase activity. The bovine and human F_1_ subcomplexes share high sequence identity (98% and 99% identity for α and β subunits, respectively), while the residues that form the AUR binding site are identical in the two species. Thus, we assume that the properties derived from the analysis of BtF_1_ structure would largely reflect those of the human ortholog. Using molecular dynamics simulations and end-point binding free energy calculations, novel aspects of the AUR binding sites were revealed regarding intra- and intersubunit communications, conformational trends, hot spot binding residues, and solvent sites that could be useful as pharmacophoric guides in virtual screening campaigns. In addition, analysis of bacterial sequences provided information on the conservation of the identified hot spot residues. This information could be relevant for the search for inhibitory molecules of this enzyme to treat human diseases or hinder the life cycle of pathogens.

## 2 Methods

**Molecular dynamics simulations.** MD simulations were performed with the AMBER 16 suite and the FF14SB force field (Maier et al., 2015; Case et al., 2016) using the crystal structures of bovine mitochondrial F_1_-ATPase solved in the ground state (PDB ID: 2jdi (Bowler et al., 2007)) and complexed with AUR B (PDB ID: 1cow (van Raaij et al., 1996)). In both F1 structures, the first 8 to 23 residues of the α and β subunits are missing, and the β subunits have not solved up to four residues at the C-terminus. To avoid adding artificial terminal charges, the first and last solved residues were capped with ACE and NME groups, respectively. In the ground state structure, segments 402–409 and 388–396 of B (α_TP_) and E (β_E_) chains, respectively, are not solved. These residues, corresponding to the turn segment of the HTH motif in each subunit, were modeled as loops using Modeller v9.20 (Šali et al, 1993). The γ subunit segments 45–76 and 87–208 are not solved in both F_1_ structures. Hence, only residues 1–44 and 209–272, corresponding to the two large α-helices were included in the simulations. These helices remained stable along all trajectories, and the regions embedded in the catalytic hexamer maintained their conformation practically invariant with respect to the crystallographic structure (RMSD <0.5 Å over the backbone heavy atoms between the average MD and crystal structures). Simulations of the AUR complexes with F_1_ from *E. coli* (PDB ID: 3oaa (Cingolani and Duncan, 2011)) and *Mycobacterium smegmatis* (PDB ID: 6foc (Zhang et al., 2019)) were also performed. AUR was docked on the crystal structures of these bacterial enzymes using BtF_1_-AUR complex as a template. The conformations of the side chains exhibiting steric clashes with the inhibitor were modified to fit the closest conformation observed in BtF_1_, using the Dunbrack 2010 rotamer library (Shapovalov and Dunbrack, 2011). In the case of missing residues, the corresponding terminal residues were blocked similarly to BtF_1_. In the mycobacterium enzyme, the missing segments 42–46 and 109–113 of the three β subunits, both in the N-terminal domain, were modeled. Three 1-μs replicas for each system were run, yielding a total simulation time of 12 μs. Proteins were protonated at pH 7.4 using PDBfixer (Eastman et al., 2017). All nucleotides and Mg^2+^ ions were included. ATP and ADP parameters were taken from (Meagher et al., 2003). Using AMBER’s tLeap, each protein was placed in a truncated octahedral box, initially spanning 12 Å further from the solute in each direction, and solvated using the TIP3P water model (Jorgensen et al., 1983). The hydrogen mass repartitioning scheme was implemented using the ParmEd (https://github.com/ParmEd/ParmEd), which allows the use of a 4 fs time step integration (Hopkins et al., 2015). The system was geometrically optimized using the steepest descent algorithm for 5,000 cycles to adjust the orientation of the solvent and remove local clashes. Initial velocities were assigned to get a 150 K distribution, slowly increasing the temperature up to 310 K in 0.8 ns in the NVT ensemble. The system was further equilibrated for 1 ns at 310 K in the NPT ensemble. Production was run in the NPT ensemble. Systems were simulated using periodic boundary conditions and Ewald sums (grid spacing of 1 Å) as implemented in the PMEMD module (Salomon-Ferrer et al., 2013) for treating long-range electrostatic interactions with a 9 Å cutoff for direct interactions (Darden et al., 1993). The same cutoff was applied to Lennard-Jones interactions. Temperature and pressure control was achieved using the Langevin thermostat with a collision frequency of 4 ps^-1^ and the Berendsen barostat with a pressure relaxation time of 2 ps, respectively. The SHAKE algorithm was enabled to fix any bond involving hydrogen atoms (Ryckaert et al., 1977; Miyamoto and Kollman, 1992). Unless otherwise stated, no other constraints were used. Coordinates were saved every 10 ps AUR B topology and parameter files were generated with the antechamber suite and the general Amber force field (GAFF2) for organic molecules using AM1-BCC atomic charges (Wang et al., 2004). AUR parameters used are listed in Supplementary Table S1 in the *Supplementary Material*. Using the backbone heavy atoms, trajectory conformers were superimposed on the crystal structure of each AUR-binding site. Otherwise, the use of larger portions of the protein (e.gr., complete F_1_) introduced minor, although significant, local variations in the position of the binding residues. Trajectory analysis was performed with CPPTRAJ (Roe and Cheatham, 2013) and Chimera UCSF v16 (Pettersen et al., 2004) software packages. Protein structure representations were generated with ChimeraX v1.3 (Pettersen et al., 2021).

**Aurovertin binding sites**. AUR-binding residues at β_E_ and β_TP_ sites were defined as those within 5 Å from the inhibitor in the crystal structure of the BtF_1_ complex. In both pockets, the same set of binding residues was found: βA^338^, βI^339^, βL^342^, βI^344^, βP^350^, βL^351^, βL^378^, βQ^379^, βY^381^, βK^382^, βQ^385^, βQ^411^, βR^412^, βE^454^, βQ^455^, βY^458^, βK^469^. αE^399^ was additionally found at the β_TP_ binding site.

**Cross-correlation analysis:** The description of the dynamic movement of the system atoms and the extent of the dynamic correlation was calculated as a covariance between the pairwise fluctuations (Estabrook et al., 2005). The cross-correlation coefficient *c(i,j)* was calculated using the following equation:

$$c\left(i,j\right)=\frac{\langle \Delta r_{i}∙\Delta r_{j}\rangle }{{\langle \Delta r_{i}^{2}\rangle }^{1/2}\;{\langle \Delta r_{j}^{2}\rangle }^{1/2}}$$ (1)

where *Δr_i_* and *Δr_j_* are the ensemble average displacement vectors of atoms *i* and *j*, respectively. Positively correlated motion implies that two atoms move in the same direction, whereas an anticorrelated motion implies the atoms move in the opposite direction. (Ichiye and Karplus, 1991).

**Dihedral angle principal component analysis**. Principal component analysis (PCA) is employed in MD as a data dimensionality reduction technique that converts a set of correlated motions into a set of orthogonal principal components containing the dominant trend of the protein’s collective motions (Stein et al., 2006). To correctly separate the overall and internal motions (Altis et al., 2008), dihedral angle PCA (dPCA) was performed on the AUR binding site residues on the concatenated trajectories of each system, using CPPTRAJ (Roe and Cheatham, 2013). These angles were transformed to a linear metric coordinate space to avoid circularity problems, using the functions sin(x) and cos(x). Then, the 2N covariance matrix was calculated. The next step was the decomposition (diagonalization) of the covariance matrix, where the instantaneous linear correlations between variables were removed and the eigenvectors and eigenvalues were computed. The eigenvalues were arranged in a descending order.

Two-dimensional free energy landscapes (FEL) with the two first principal components, PC1 and PC2, were built with PyEMMA (Scherer et al., 2015), using the following equation:

$$F\left(x\right)=-k_{B}*T*\ln P\left(x\right)$$ (2)

where *F(x)* is the *x*-coordinate free energy landscape, *k_B_* is the Boltzmann constant, *T* is the absolute temperature and *P(x)* is the *x*-coordinate probability distribution, taken as the two-dimensional histogram of PC1 and PC2.

**Markov State Models (MSM)**. To quantify the relative abundance of visited conformations, MSM were constructed with PyEMMA (Scherer et al., 2015). As a first step, dPCA was applied over the MD trajectories. The resultant subspace of the first two principal modes was then discretized by creating a set of 1,000 conformational microstates using the *k*-means clustering method. The MD trajectories were encoded as a stochastic matrix based on the transition probabilities constructed using maximum likelihood and Bayesian estimation. Perron-cluster cluster analysis (Deuflhard and Weber, 2005) was implemented to group microstates into macrostates based on kinetic similarities.

**Solvent-site identification and guided docking.** Solvent sites for ethanol were determined using the MDMix method, as described elsewhere (Alvarez-Garcia and Barril, 2014; Avila-Barrientos et al., 2022). Three replicas of 20 ns each were run using the AUR-bound crystal structure, from which the two inhibitor molecules were removed. Conditions similar to those mentioned in the MD section were used, but with the following differences: the protein was embedded in a box of water/ethanol 80/20% v/v, and Cartesian restrictions of 0.1 kcal/mol A^2^ were applied on the protein heavy atoms. After trajectories were aligned, density maps for probe atoms were obtained by building a static mesh of grids over the entire simulation box and counting the appearance of probe atoms in each grid during the trajectory. The observed appearance was converted into binding free energy (*ΔG*_*SS*_) applying the Boltzmann relationship, considering the observed probe atom distribution and the expected distribution in bulk solvent at 1.0 M. Solvent sites were filtered by imposing an energy threshold of 1 kcal/mol. Solvent sites were used as pharmacophoric elements to dock AUR and related compounds at the AUR binding site using rDock (Ruiz-Carmona et al., 2014), as described elsewhere (Avila-Barrientos et al., 2022). Ligand protonation states were generated with Open Babel v. 2.3.1 (O’Boyle et al., 2011).

**Relative binding free energy prediction.** Relative binding free energies for the complex (*G_PL_*) and the free reactants (*G_P_*, *G_L_*) were calculated using the MM-PBSA (Molecular Mechanics Poisson-Boltzmann Surface Area) single-trajectory approach (Wang et al., 2019). No convergence was observed using the two or three-trajectory approaches. Free energy changes (*ΔG_PB_*) and their decomposition per binding residue were calculated with the MMPBSA. py script (Miller et al., 2012), according to:

$${∆G}_{PB}=\langle G_{PL}-G_{P}-G_{L}\rangle =∆H-T∆S={∆E}_{MM}+{∆G}_{solv}-{T∆S}_{conf}$$ (3)

$${∆E}_{MM}={∆E}_{bond}+{∆E}_{ele}+{∆E}_{vdW}$$ (4)

$${∆G}_{sol}={∆G}_{PB}+{∆G}_{np}$$ (5)

$${∆G}_{np}=\gamma *SASA+b+E_{disp}$$ (6)

where *ΔE*_*MM*_ is the potential energy change in vacuum, *ΔG*_*solv*_ is the solvation energy, and *TΔS*_*conf*_ is the conformational entropy change. *ΔE*_*MM*_ is further divided into bonded (*ΔE*_*bond*_), electrostatic (*ΔE*_*ele*_) and van der Waals (*ΔE*_*vdW*_) energies changes. *ΔG*_*solv*_ is the sum of the polar solvation contribution, calculated using the Poisson-Boltzmann model, and the non-polar contribution (*ΔG*_*np*_), calculated as the sum of the free energy of the cavity and the van der Waals interactions between the solvent and the solute as a linear relationship to the solvent-accessible surface area (*SASA*). Smooth surface (sasopt = 2) was used, with surface tension γ and offset correction *b* values of 0.0378 kcal/molÅ2 and 0.5692 kcal/mol, respectively (Ye et al., 2010). *E*_*disp*_ is the attractive interaction between the solute and the solvent (Tan et al., 2007). The atom-type/charge-based radii were used (Tan et al., 2006). *T*Δ*S* contains the entropies arising from the particle number change (TΔS_r-t_) and the freezing of rotatable bonds (TΔS_conf_). The first contribution has a penalty of ∼3 kcal/mol (Amzel, 1997; García-Hernández and Hernández-Arana, 1999); the second is usually calculated with the normal mode analysis or the quasi-harmonic approximation (Chang et al., 2005; Wang et al., 2019), although this term was not calculated in this work because of the large system size. The calculations were performed with 400 frames spaced every 6 ns. Dielectric constants for solvent and solute were set to 80 and 2, respectively, using an ionic strength of 150 mM. The required topology files were created using the ante-MMPBSA.py script implemented in AmberTools (Case et al., 2016).

**Sequence analysis of the AUR binding site.** Bacterial ATP synthase β subunits sequences were retrieved from the UniProt database (Bateman et al., 2021). Jalview2 (Waterhouse et al., 2009) was used to curate the database, excluding redundant sequences (identity <100%). The curated database was used to generate a multiple sequence alignment with Clustal Omega (Sievers et al., 2011). Sequence logos were generated using the Weblogo3 server (Crooks et al., 2004). Virtually the same results were obtained using 98% or 99% redundancy sequence identity cutoffs.

## 3 Results

**Protein-inhibitor interaction patterns**. To carry out a computational characterization of the interaction of F_1_ with AUR B (AUR^+^), we used the crystal structure of the bovine subcomplex bound to two inhibitor molecules (van Raaij et al., 1996). As the reference structure of the subcomplex without AUR B (AUR^─^), the so-termed “ground state” structure was used (Bowler et al., 2007). This structure has the particularity of being solved bound to nucleotides but in the absence of any inhibitor, so it is considered an accurate representation of the catalytic intermediate in the direction of hydrolysis. The AUR-bound and “ground state” structures of BtF_1_, like most of those experimentally solved so far, correspond to the catalytic dwell in a 120° rotary substep (Sobti et al., 2021). Therefore, the following analysis circumscribed to the study of the conformational behavior of this metastable state.

To characterize the dynamics of the protein-inhibitor interactions, three MD replicates of 1 µs each were run for BtF_1_ bound to the two AUR B molecules, a time span that is two to three orders of magnitude smaller than the actual enzyme rotation time length (Sekiya et al., 2017). An autocorrelation analysis of the backbone dihedral angles indicated that relaxation of both binding sites occurred within the first 0.2 µs of simulation (Supplementary Figure S1A). Faster convergence was observed for RMSD (Supplementary Figure S1B). Therefore, the analysis was performed on a concatenated trajectory of the last 0.8 µs of each replica. Similar interaction patterns with the inhibitor were observed in β_TP_ and β_E_ (Figure 2C and Supplementary Figure S2). βQ^379^, βE^454^ and βK^469^ lost interaction with the inhibitor during the simulation (gray residues in Figure 2B), with cumulative contact frequencies <0.25 (Figure 2C). Therefore, these residues were excluded from further analysis. The other 14 β-subunit residues showed cumulative contact frequencies >0.8. A large conformational variability was observed in α_TP_. In two trajectories, HTH of this subunit approached AUR B, and four additional residues (αL^392^, αE^393^, αA^395^, αQ^396^) persistently contacted the inhibitor (Figures 2B,C). In the other trajectory, HTH stayed away from AUR, and even the interaction with αE^399^ was completely disrupted. On the average, each of these four α_TP_ residues had a cumulative contact frequency >0.5, thus, together with αE^399^, they were considered AUR-binding residues.

**FIGURE 3:**
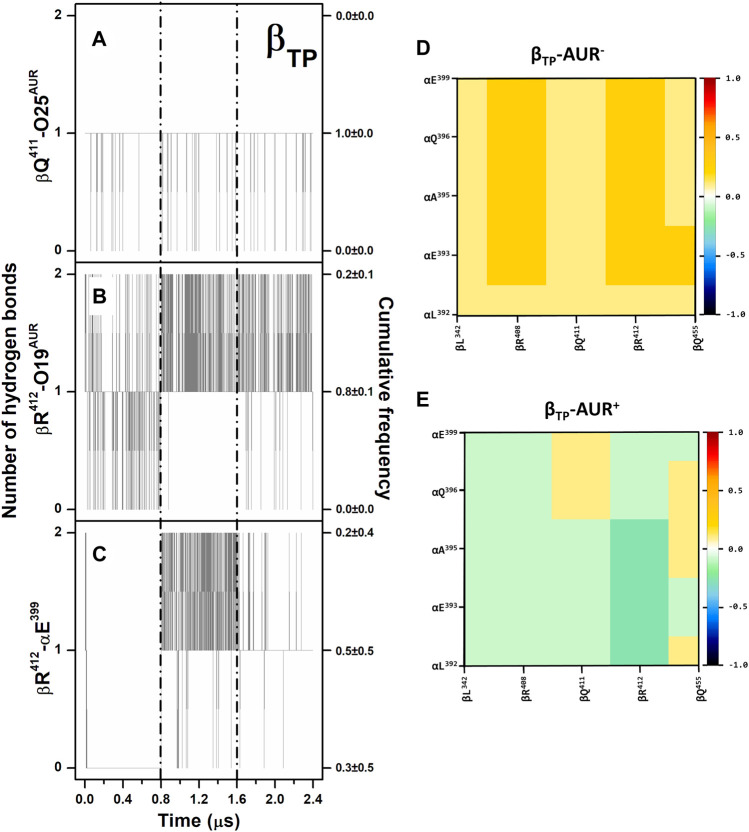
Hydrogen bonding in β_TP_-AUR+ and correlated motions of α_TP_ and β_TP_ CTD residues. **(A and B)** Protein-inhibitor hydrogen bonds. **(C)** α_TP_-β_TP_ hydrogen bond. Data for the three concatenated 1-µs replicas are shown, after subtracting the first 0.2 µs of simulation from each one. The trajectories of each replica are delimited by dashed lines. Cumulative frequencies refer to the total fraction of time each number of hydrogen bonds was observed in the simulations. **(D and E)** Dynamic cross-correlation maps of α_TP_ and β_TP_ CTD residues, calculated over side chain atoms around their mid-positions for 2.4-μs MD simulations in the absence and presence of AUR.

Overall, the poses and interaction modes of AUR B observed in the crystal structure were kept at both binding sites during the MD trajectories. The hydrogen bonds between βR^412^-O19 and βQ^411^-O25 remained formed throughout the simulations, except at the β_E_ site, where the βQ^411^-O25 bond was broken in the last 0.3 µs of one trajectory because of a partial ligand dissociation (Figures 3A,B and Supplementary Figure S3A,B). Recurringly, αE^399^ and βR^412^, which are 8–10 Å from each other in the crystal structures, formed a salt-bridge at the β_TP_ site (Figure 3C). This contact, either mono or bidentate, was present ∼70% and ∼90% for AUR^+^ and AUR^─^, respectively. The interaction between βQ^455^ and αE^399^ was observed ∼60% of the time for AUR^─^, whereas the inhibitor completely blocked this interaction. Although sporadically (∼2%), the interaction between βR^408^ and αE^399^, residues that are ∼10 Å from each other in the crystal structure, was only observed for AUR^─^. This hydrogen-bond network transiently occluded the β_TP_ site, generating a steric hindrance for AUR access. Interestingly, βR^408^, βR^412^, βQ^455^, and αE^399^ are part of a cooperative hydrogen bond network (which includes βL^342^ and αQ^396^) that stabilizes the interaction between CTD of α_DP_ and β_DP_, as illustrated in Supplementary Figure S3C. Consistent with this, a dynamic cross-correlation analysis indicated AUR decouples the side chain movements of the residues that form this network, as shown in Figures 3D,E. Thus, these results suggest that α_TP_ and β_TP_ exhibit a clear trend to interact with each other towards the adoption of an α_DP_/β_DP_-like conformation. The importance of this hydrogen-bond network has been demonstrated through directed mutagenesis experiments, showing that its disturbance affects the catalytic activity and can even prevent the assembly of the enzyme (Mao et al., 2008). These mutations do not change the affinity for the nucleotides (Ichikawa et al., 2005; Mao et al., 2008). Therefore, AUR B seems to hinder the interaction between the CTD of α_TP_ and β_TP_, suggesting that the action of this inhibitor is partially related to the interruption of the dynamic communication of conformational changes between these subunits. Consistent with this, inhibitors like IF1, ε, ζ, or glycomacrolides prevent the formation of the CTD interactions established at the α_DP_ and β_DP_ interface (Cabezón et al., 2003; Cingolani and Duncan, 2011; Morales-Rios et al., 2015; Reisman et al., 2022).

**FIGURE 4:**
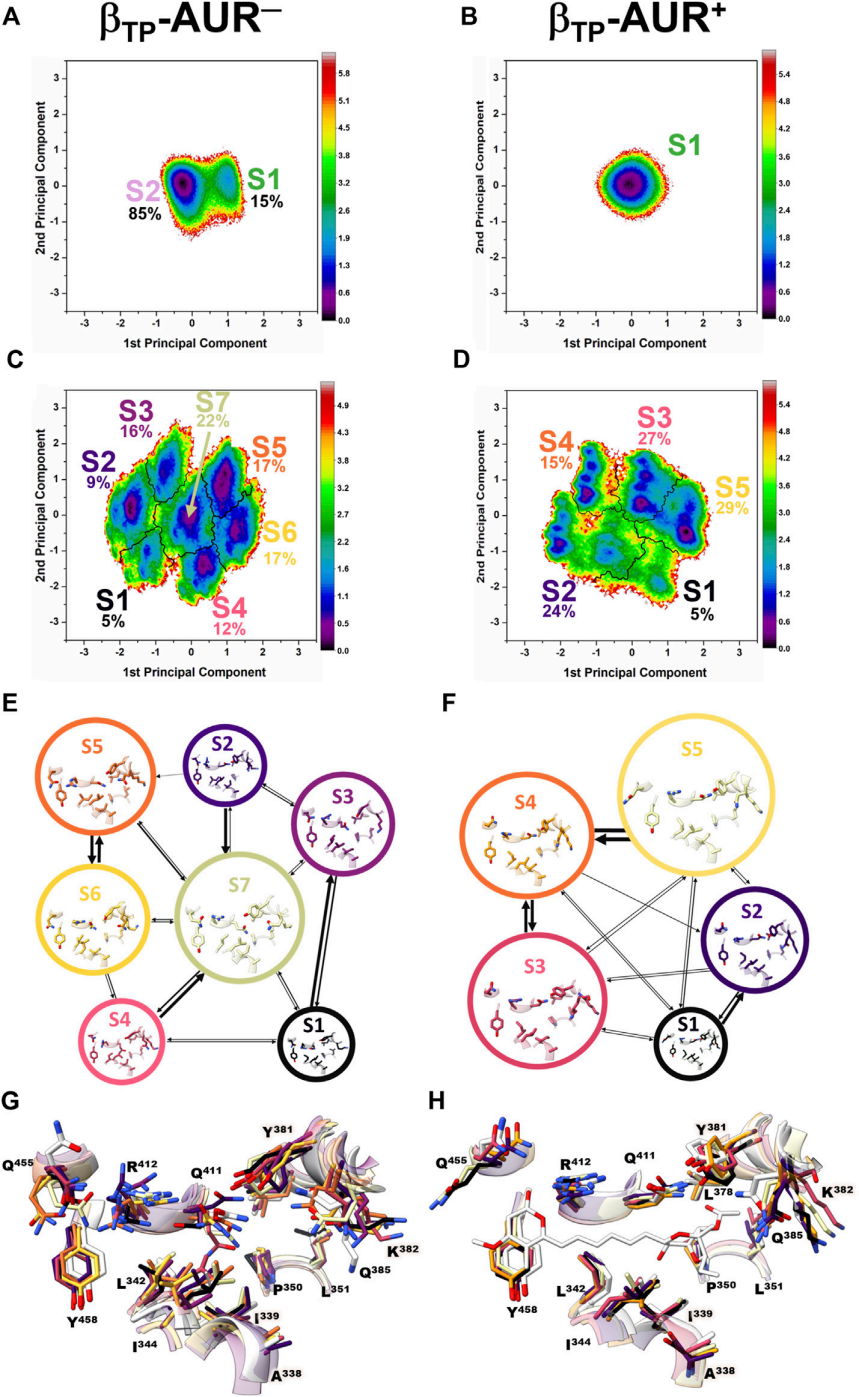
Dihedral angle free energy landscapes (FEL) for the AUR binding site residues in β_TP_ **.** FEL (in *k*_*B*_T units) were obtained from a dPCA projected onto the first two principal components in the absence (left column) and presence (right column) of the inhibitor. **(A and B)** Backbone dPCA. One and two metastable conformational states were observed for AUR^+^ (S1) and AUR^─^ (S1, S2), respectively. The percentage of cumulative frequency is indicated. The main difference between S1 and S2 was the ψ angle value of I^344^. **(C and D)** Side chain dPCA. The black lines delimit the macrostates identified through a Markov-state model analysis. **(E and F)** Network transition pathway of the Markov-state model. The thickness of the connecting arrows is proportional to the transition probability. **(G and H)** Superimposition of representative conformations for each attraction basin in **(E and F)**. Macrostates were labeled S1, S2 and so on from lowest to highest occupancy.

**FIGURE 5:**
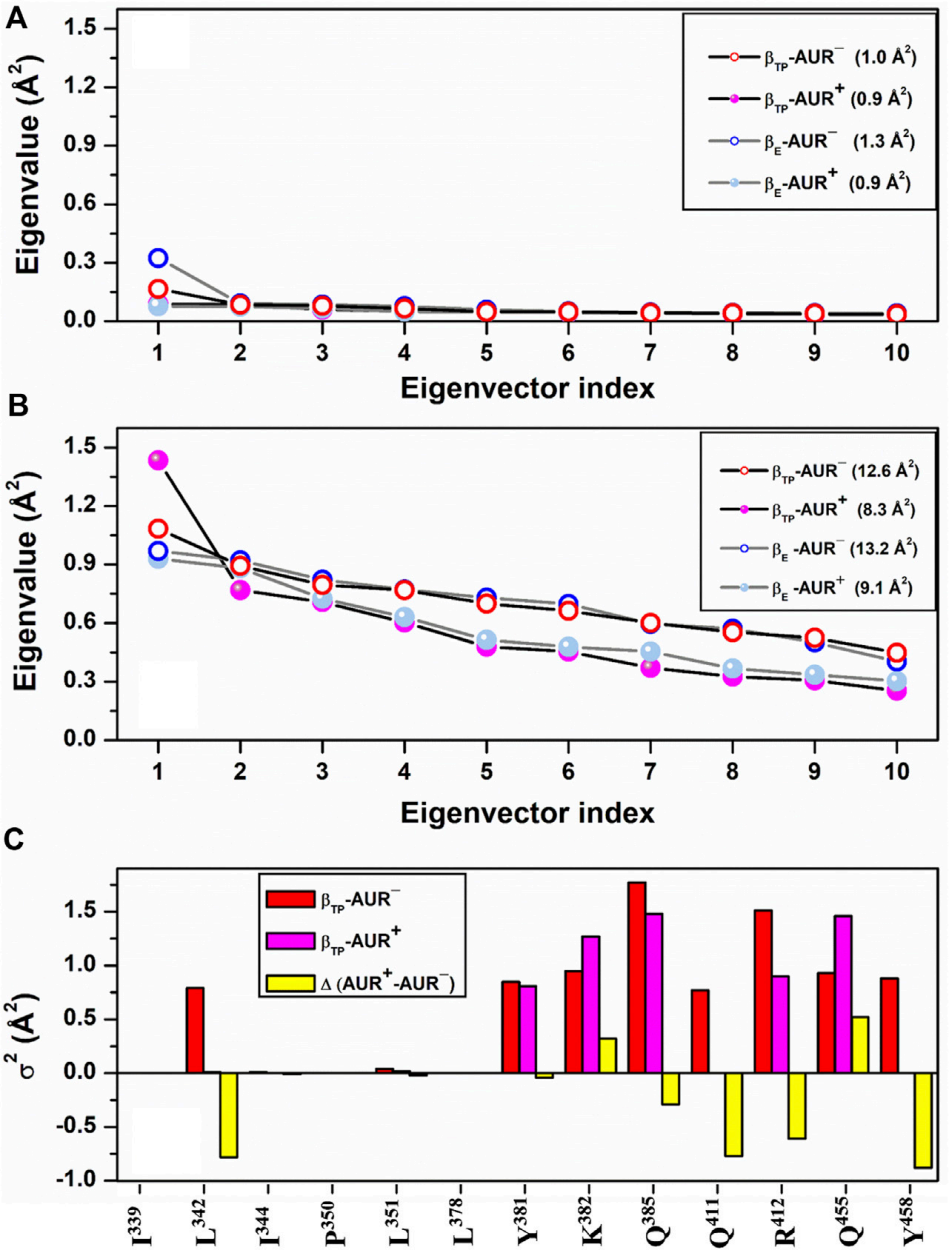
dPCA scree plots for AUR binding residues. The eigenvalue distributions for the first ten eigenvectors for backbone and side chain dihedral angles are shown in **(A)** and **(B)**, respectively. Values in parentheses indicate the total variance for the indicated system. **(C)** Cumulative variance per residue (σ^2^) of side chains at the β_TP_ site. Δ(AUR^+^─AUR^─^) is the σ^2^ difference in presence minus in absence of the inhibitor. Values correspond to 70% of the total variance.

**Conformational dynamics of the AUR binding sites.** Principal component analysis was performed on dihedral angles to assess conformational fluctuations of the AUR binding sites. Figure 4 shows the conformational landscape of the β_TP_ binding site for the first two principal modes (those capturing the largest conformational dispersion). The β_E_ results are shown in Supplementary Figure S4. An analysis of backbone dihedral angles revealed two basins of attraction (S1 and S2) for AUR^─^ that, based on an inspection of the conformers of each state, vary significantly only in the ψ angle of βI^344^ (Figure 4A). S1 adopted ψ = 120 ± 13°, a conformation like that observed in the two crystal structures of BtF_1_, and S2 distributed around ψ = 57 ± 11°. S1 was less populated than S2 in β_TP_ and nearly equipopulated in β_E_ (Supplementary Figure S4A). The inhibitor decreased the overall dynamics of both sites to a similar value (Figure 5A), populating the same single attraction basin equivalent to S1 (Figure 4B and Supplementary Figure S4B), that is, the crystal conformer. Because of the greater mobility of the ligand-free binding site in β_E_, the inhibitor induced larger backbone stiffness compared to the β_TP_ site. The five α_TP_ residues also formed a single attraction basin for AUR^+^ and three basins for AUR^─^ (Supplementary Figure S5). A side chain dihedral angle analysis revealed a more complex conformational behavior. To discern the underlying collective movements, a MSM analysis was performed. These kinetic models describe the conformational dynamics of biomolecular systems in terms of transition rates between conformational states (Pande et al., 2010; Husic and Pande, 2018). As shown in Figures 4C,D and Supplementary Figure S4C,D, the conformational landscape of the side chains at the β_TP_ binding site is composed of several attraction basins. The number of attraction basins and the total variance decreased with AUR B (Figure 5B). In β_TP_, the inhibitor completely froze the side chains of βL^342^, βQ^411^, and βY^458^, while the side chains of βI^339^, βI^344^, βP^350^, βL^351^, and βL^378^ were already frozen without the inhibitor (Figures 4E–H and Figure 5C). βQ^385^ and βR^412^ partially decreased their mobility with AUR B, and the peripheral residues βK^382^ and βQ^455^ slightly increased it. Finally, regardless of the presence of AUR, Y^381^ had the same mobility. A similar behavior was observed for β_E_ (Supplementary Figure S6). Overall, these results showed that the AUR binding site is preformed in both catalytic subunits, undergoing relatively minor rearrangements and conformational constraints upon inhibitor binding, although the reduction in mobility was somewhat greater at the β_E_ site. To contrast these results with experimental data, we analyzed the conformational variations between solved BtF_1_ structures (resolution better than 3.5 Å). As shown in Supplementary Figure S7A, 28 structures retrieved from the PDB closely overlapped each other at the AUR binding site in β_TP_ (RMSD <0.8 Å over the backbone heavy atoms). Thus, regardless of crystallization conditions, bound inhibitor, and/or the occupancies at the nucleotide binding sites, the backbone conformation at the AUR binding site is practically invariant. In addition, when projected onto the FEL for β_TP_-AUR^─^, most of the crystal structures fell within the most visited macrostate region, while two of them resembled the second largest macrostate (Supplementary Figure S7B). Finally, it is noteworthy that the shift of the α_TP_ HTH towards the AUR binding site observed in our simulations is present, although less pronounced, in four solved BtF_1_ structures in complex with IF1 (Gledhill et al., 2007b; Bason et al., 2014, 2015).

**FIGURE 6:**
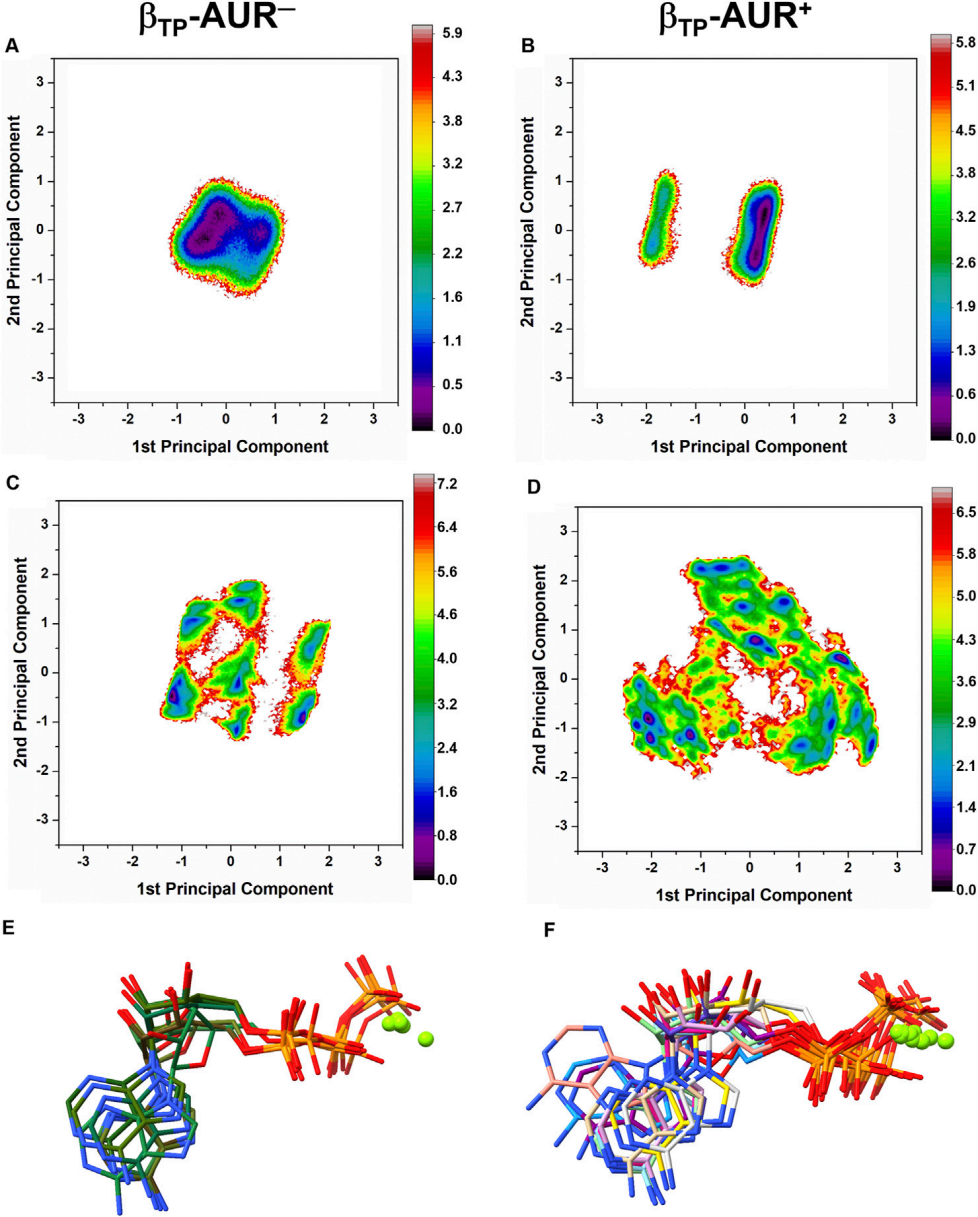
Dihedral angle free energy landscapes (FEL) for the nucleotide binding site in β_TP_. **(A)** Backbone FEL for AUR^─^. **(B)** Backbone FEL for AUR^+^. **(C)** Side chain FEL for AUR^─^. **(D)** Side chain FEL for AUR^+^. FEL (in *k*_*B*_ *T* units) were obtained from a dPCA projected onto the first two principal components in the absence (left column) and presence (right column) of the inhibitor. Residues with χ angles within 5 Å of the nucleotide in β_TP_ (V^160^-V^164^, R^189^, T^190^, R^260^, Y^311^, Y^345^, P^346^, Q^416^, F^418^, F^424^, T^425^) were included in the analysis. **(E)** and **(F)** Representative MgATP conformations for AUR^─^ and AUR^+^, respectively. Magnesium atoms are shown as green spheres.

**FIGURE 7:**
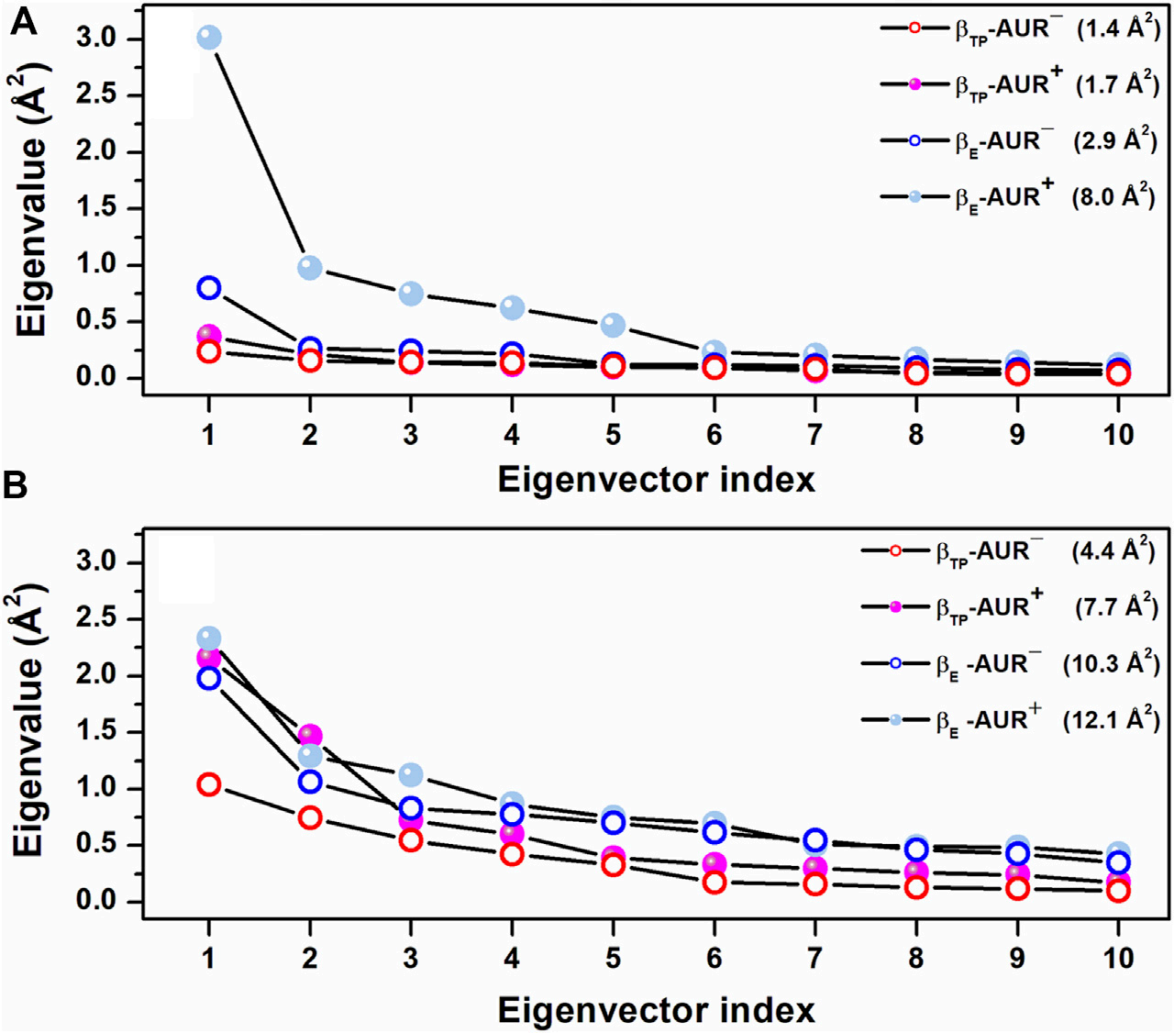
Scree plots obtained from dPCA for nucleotide binding residues in β_TP_. **(A)** Backbone dihedral angles. **(B)** Side chain dihedral angles. The eigenvalue distributions for the first ten eigenvectors are shown. The values in parentheses indicate the total variance for the corresponding system. Residues with χ angles within 5 Å of the nucleotide in β_TP_ (V^160^-V^164^, R^189^, T^190^, R^260^, Y^311^, Y^345^, P^346^, Q^416^, F^418^, F^424^, T^425^) were included in the analysis.

**Effects of AUR on catalytic sites**. *In vitro* studies have shown that AUR exerts a mild positive cooperative effect on the catalytic sites of BtF_1_ and EcF_1_ (Issartel et al., 1983b; Issartel and Vignais, 1984). Perhaps the best solved measurement of the reciprocal binding effect of AUR and substrate nucleotides was made on the isolated β subunit (Issartel and Vignais, 1984; van Raaij et al., 1996; Johnson et al., 2009). The inhibitor increases ADP/ATP affinities by 3-7-fold (∼0.4–1.1 kcal/mol of Gibbs free energy). Likewise, the AUR-like molecule citreoviridin decreases *K*_*m*_ in F_1_ from *Trypanosoma cruzi* (Cataldi de Flombaum and Stoppani, 1981). Thus, in addition to acting as a physical blocker, AUR has effects on F_1_ beyond its own binding sites. To investigate these effects, we analyzed the conformational properties of the nucleotide binding sites in β_TP_ and β_E_. Figure 6 shows the conformational landscapes for the backbone and side chain dihedral angles of the nucleotide binding residues in β_TP_. Strikingly, more complex landscapes and larger mobilities were observed with AUR B in β_TP_ (Figures 6A–D and Figure 7). Along with this, ATP mobility increased significantly for AUR^+^ (Figures 6E,F). Interestingly, even without nucleotide in the catalytic site, an increased mobility in β_E_ was observed for AUR^+^ compared to AUR^─^ (Figure 7 and Supplementary Figure S8). Overall, these results are consistent with a favorable conformational entropy contributing to the increase in nucleotide affinity elicited by the inhibitor.

**Solvent site identification and free energy calculations.** To investigate the energetic relevance of AUR binding site residues, we performed MD simulations in an ethanol/water solvent box using the MDMix method (Alvarez-Garcia and Barril, 2014). In brief, this approach allows the organic solvent to diffuse unbiasedly over the protein surface through a process governed only by the time evolution of the atomic energies and degrees of freedom of the system. Those regions of the protein that interact favorably with the cosolvent will define solvent sites (SS) characterized by higher occupancies than in the bulk solvent, from which it is possible to calculate the free energy of interaction (*ΔG*_*SS*_) at each site. Active and allosteric sites usually define SS, since they have the appropriate stereochemistry to form stable interactions with organic molecules (Alvarez-Garcia and Barril, 2014; Arcon et al., 2017). MDMix has been valuable not only in helping to determine the dominant residues or “hot spots” in molecular recognition (Arcon et al., 2017), but also in guiding the discovery and design of new pharmacological molecules, both orthosteric and allosteric (Rachman et al., 2020; Talibov et al., 2021; Avila-Barrientos et al., 2022).

**FIGURE 8:**
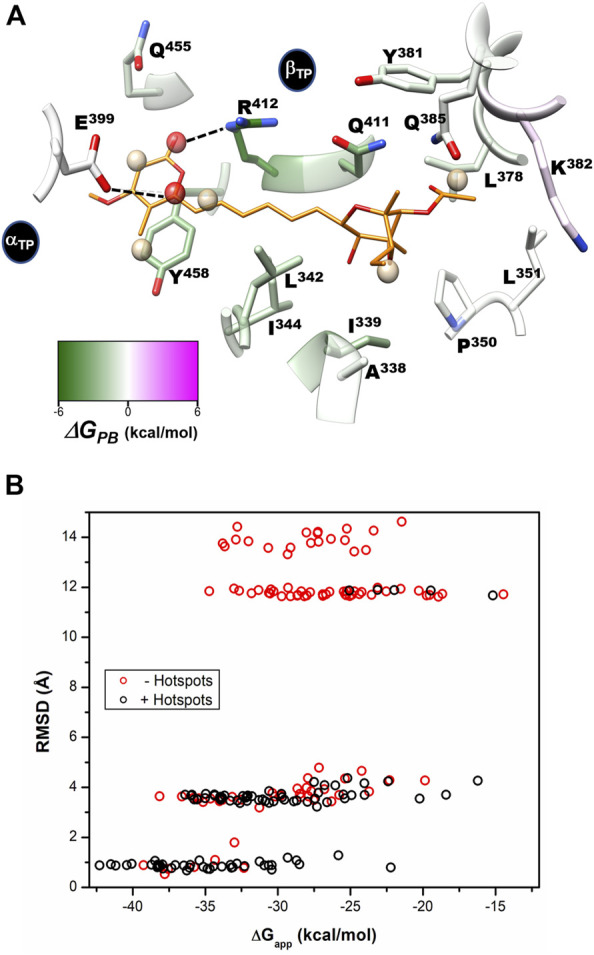
Per-residue free energy decomposition, solvent site identification, and guided docking. **(A)** Per-residue decomposition of the binding free energy (*ΔG*_*PB*_) calculated with the MMPBSA method. Residues that favor interaction with the inhibitor are shown in green. Identified hydrophobic (SS_HP_) and hydrophilic (SS_POL_) solvent sites are shown as tan and red spheres, respectively. The five SS_HP_ overlapped with AUR apolar carbons, while one SS_POL_ reproduces the polar-to-polar interaction between βR^412^ and AUR O19. The other detected SS_POL_, which interacts with αE^399^, does not have an equivalent interaction with AUR. CHEWD was used to generate the image (Raza et al., 2019). **(B)** AUR-docking score (*ΔG*_*app*_) obtained with rDock guided (black symbols) and not guided (red symbols) with solvent sites (hot spots) as pharmacophores. 100 docking runs were carried out for each instance. RMSD of each docked pose was calculated against the crystallographic pose of the inhibitor.

As revealed from the trajectories without the inhibitor, the AUR binding site tended to be occluded because of the propensity to form hydrogen bonds between α_TP_ and β_TP_ residues. Therefore, to characterize the solvation of the conformation competent to bind the inhibitor, harmonic constraints were applied to all protein heteroatoms. Figure 8 shows SS determined for the β_TP_ site from the average of three independent trajectories, each 20 ns long. Two types of SS, one for the methyl group and another for the hydroxyl group of ethanol, were calculated as probes for hydrophobic (SS_HP_) and polar (SS_POL_) interactions, respectively. βA^338^, βI^339^, βL^342^, βI^344^, βL^351^, βL^378^, βY^381^, βK^382^, βR^412^, βQ^455^ and βY^458^, all of them involved in AUR binding, stabilized the five SS_HP_ detected (Figure 8A). Three SS_HP_ were observed in the pyrone binding region and another two in the bicyclo region of AUR. βY^458^ simultaneously stabilized three SS_HP_, highlighting the importance of this residue in the interaction with organic molecules. αE^399^ and βR^412^ stabilized the two detected SS_POL_. The one near αE^399^ did not reproduce any equivalent interaction with AUR B. In contrast, the other SS_POL_ replicated the interaction between βR^412^ and O19 of AUR B, whereas no SS_POL_ was solved for the βQ^411^-O25 interaction. Interestingly, molecular docking experiments on BtF_1_ guided by all six SS emulating the interaction with AUR B (i.e., excluding the one close to αE^399^) improved the ability to predict the crystal position of the inhibitor (Figure 8B). In the absence of SS information, the docking success rate for predicting the correct AUR B position was only 39%. Relative to the crystal binding mode, the pyrone appeared inverted in most poses. In contrast, the success rate increased to 91% with the use of SS information. Similar results were observed with citreoviridin and asteltoxin, two AUR-like compounds (data not shown). Less SS were reproduced at the β_E_ site. The two SS_POL_ in β_TP_ were not solved (Supplementary Figure S9), suggesting the importance of the α subunit in defining the interaction.

To assess the per-residue energy contribution in the interaction with AUR B, the binding free energy (*∆G*_*PB*_) was calculated using the MMPBSA method (Wang et al., 2019). This approach combines molecular mechanics and a continuum solvation model to calculate the endpoint binding free energies (Equation 3) (Genheden and Ryde, 2015). The energy of the solute is calculated by the force field. Solvation free energy has two components, polar and nonpolar solvation energies (Equation 5). The first term is calculated by solving the Poisson-Boltzmann equation. In a broad sense, this means calculating the energy associated with transferring charges from the vacuum to the continuum. The second models the solute cavity formation and the non-electrostatic interactions between the solute and the continuum close to the cavity. This is usually calculated with an approximation based on surface area and a van der Waals dispersion term. In the single-path approach, structural changes associated with ligand binding are not considered, and conformational entropy is generally ignored, as it does not improve calculations (see further details in the Methods section) (Wang et al., 2018).

**FIGURE 9:**
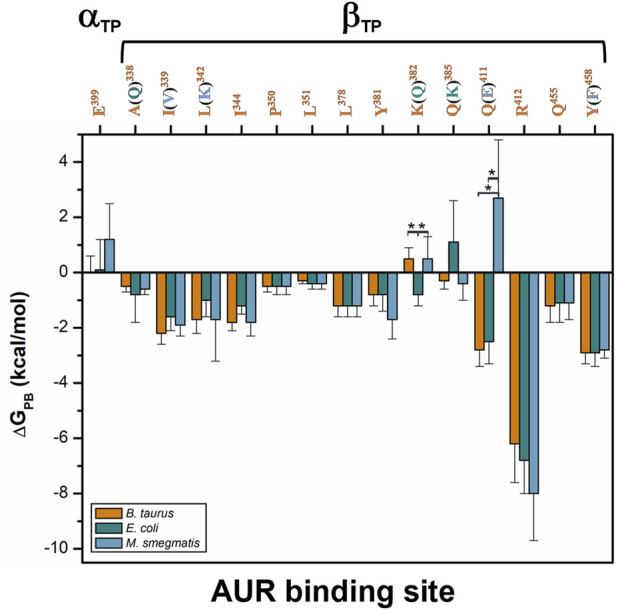
Per-residue free energy comparison of the AUR binding site of bovine and two pathogen species. The numbers correspond to BtF1. Residue variations relative to BtF_1_ are in parentheses. Residues with statistically significant energy differences (α < 0.05) according to a Student-t distribution are marked with *.

Based on reported *K*_*d*_ values, the experimental binding Gibbs free energy (*ΔG*_*b*_) for AUR B with BtF_1_ is 9.5 kcal/mol (Hong and Pedersen, 2008). Using *ε* = 2, *∆G*_*PB*_ values of -18.6 ± 4.6 and -16.3 ± 3.1 kcal/mol were obtained for the β_TP_ and β_E_ sites, respectively. These magnitudes are in reasonable agreement with the experimental one, considering the loss of roto-translational (*TΔS* ∼ -3 kcal/mol (Amzel, 1997; García-Hernández and Hernández-Arana, 1999)) and conformational (*TΔS* ∼0.5 kcal/mol per frozen rotatable bond) entropies, which were not included in the calculations. In contrast, with *ε* = 1, *∆G*_*PB*_ = 9.0 ± 4.7 and 7.1 ± 4.5 kcal/mol were obtained for the β_TP_ and β_E_ sites, respectively, which clearly underestimate the experimental binding strength. Regardless of the value used for the internal dielectric constant, the per-residue energy ranking remained constant. According to the results shown in Figure 9, the residues that most contributed to the binding free energy in β_TP_ were βR^412^>βY^458^≈βQ^411^>βI^339^>βL^342^≈βI^344^>βL^378^≈βQ^455^, which ranged between -6.2 ± 1.4 (βR^412^) to 1.2 ± 0.6 (βQ^455^) kcal/mol (*ε* = 2). Similar *∆G*_*PB*_ results were obtained for β_E_ (Supplementary Figure S9). These eight residues, about half of the AUR binding site, can be considered as “hot spots” that primarily determine the affinity for the inhibitor. According to these calculations, the subsites to which the pyrone and bicyclo moieties bind contributed similarly to the overall affinity, while the linker region had a negligible contribution. Consistent with our results, *Bacillus* species such as *B.* PS3 and *B. firmus* OF4 are naturally insensitive to this molecule, which has been attributed to the substitution of βR^412^ and βY^458^ by F and R, respectively (Saishu et al., 1983; Hicks and Krulwich, 1995). In contrast, AUR inhibits the F_O_F_1_-ATP synthases of *E. coli*, *Rhodospirillum rubrum*, *Alcaligenes faecalis,* and *Paracoccus denitrificans* (Satre et al., 1979)*.* The enzymes from these bacterial species retain the same set of eight BtF_1_ hot spots for AUR binding (Figure 9). Mutational studies aimed at understanding the molecular recognition of AUR by F_O_F_1_-ATP synthase are scarce. The substitution of the equivalent bovine βR^412^ in *E. coli* by Cys, His or Trp makes the enzyme insensitive to AUR, whereas these mutations elicit a loss of catalytic activity between 0 and 29% (Lee et al., 1989, 1991).

**Comparison of binding energetics with bacterial enzymes**. To compare the AUR B binding energetics of BtF_1_ with that of pathogenic bacteria, *ΔG*_*PB*_ for the F_1_ sectors of *E. coli* (EcF_1_) and *M. smegmatis* (MsF_1_) were calculated. The crystal structures of these bacterial enzymes have not been solved in complex with AUR (Cingolani and Duncan, 2011; Zhang et al., 2019). Thus, AUR B was docked on these enzymes using the bovine complex as a template. Three MD replicates of 1 µs each were performed for each bacterial system. The AUR binding sites of EcF_1_ and MsF_1_ have 79 and 71% sequence identity with BtF_1_, respectively. Residues A^338^, K^382^, and Q^385^ in BtF_1_ are replaced by Q, Q, and K in EcF_1_, respectively, while residues I^339^, L^342^, Q^411^, and Y^458^ in BtF_1_ are replaced by V, K, E, and F in MsF_1_, respectively. Despite these changes, the energy profiles of the three species were similar to each other. (Figure 9) The K^382^Q and Q^385^K mutations between BtF_1_ and EcF_1_ exhibited almost complete compensatory effects, while the A^338^Q substitution produced no significant changes, which is consistent with the fact that the interaction with the inhibitor was mainly mediated by the C_β_ atoms of both residues. Experimental *K*_*d*_ values show that AUR binds to BtF_1_ and EcF_1_ with similar strength (Issartel et al., 1983a; 1983b), which is consistent with the overall *ΔG*_*PB*_ values obtained herein (*ΔG*_*PB*_ values of 18.6 ± 4.6 vs. 16.6 ± 4.4 kcal/mol, respectively). Therefore, the AUR binding site appears unsuitable for designing molecules that can discriminate between EcF_1_ and the mammalian enzyme. As for MsF_1_, it has not been experimentally determined whether AUR (or any other related compound) inhibits it. *ΔG*_*PB*_ did not indicate energetic changes for the mutations I^339^V, L^342^K, and Y^458^F, showing the formation of equivalent hydrophobic contacts with the inhibitor in both enzymes. In fact, the polar moieties of K in MsF_1_ and Y in BtF_1_ did not contact AUR B. The largest energy variation was observed for the Q^411^E substitution. This behavior seems to arise from the loss of the hydrogen bond between Q^411^ and AUR B O25 in BtF_1_, and the generation instead of an electrostatic repulsion with the glutamate carboxylate group in MsF_1_. To minimize this repulsion, the AUR B bicyclo rotated during the simulations to allow O25 to be more exposed to the solvent. In agreement with this, a smaller *ΔG*_*PB*_ value of -12.8 ± 5.0 kcal/mol was obtained for MsF_1_. In our simulations, AUR B remained at the β_TP_-AUR site throughout the trajectories, while rapidly dissociating from the β_E_ site (data not shown). With these results, a weaker affinity of MsF_1_ for AUR B could be hypothesized. Potentially, compounds could be sought that take advantage of the Q^411^E mutation to specifically recognize this bacterial enzyme.

**FIGURE 10:**
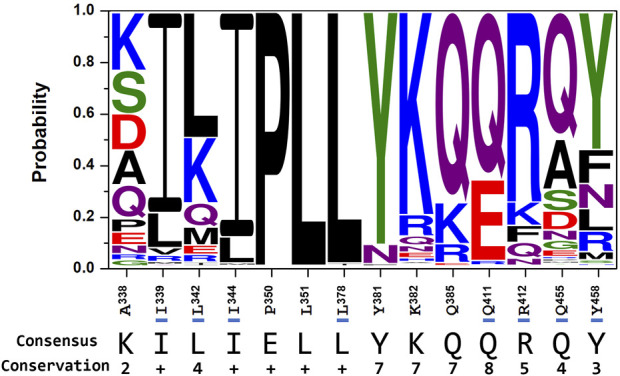
Conservation of the AUR binding site in bacterial FOF1-ATP synthases. Residues (and numbering) on the *x*-axis correspond to the bovine sequence. Residues that, based on *ΔG*_*PB*_ calculations in Figure 9, contribute one or more kcal/mol of favorable binding free energy in BtF_1_ are underlined in blue. The consensus sequence is also shown. The **Conservation** row corresponds to a scale ranging from 0 (null conservation) to 10 (= +, complete conservation of physicochemical properties of the amino acid group) as defined in (Livingstone and Barton, 1993). Multiple sequence alignment of 23,125 entries was performed with Clustal Omega (Sievers et al., 2011). Logos were generated using the Weblogo3 server (Crooks et al., 2004).

Given the potential use of the AUR binding site as an antimicrobial target, we explored its sequence conservation in the Bacteria domain. Figure 10 shows a sequence logo built from 23,125 bacterial β-subunit sequences retrieved from the UniProt database. Only 3,409 sequences (∼15%) contained the same eight residues that contribute most to AUR B affinity in BtF_1_, including human pathogens from the genera *Coxiella, Escherichia, Haemophilus*, *Legionella*, and *Rickettsia*. While 12,511 sequences (∼54%) have both R and Y/F at positions 412 and 458 of BtF_1_, respectively, only 1,497 (∼6%) sequences have these residues swapped. Based on experimental evidence, pathogenic species from genera *Staphylococcus*, *Streptococcus* and *Enterococcus*, which contain the latter sequences, should be insensitive to AUR, as proven in *Bacillus* PS3. Also, 7,297 (∼32%) sequences, including species from genera *Brucella*, *Campylobacter*, *Clostridium*, *Corynebacterium*, *Helicobacter*, *Mycobacterium*, and *Vibrio,* have E instead of Q at position 411. Hence, just considering these three residue positions, ∼6–∼48% of the sequences would have an abolished or weakened affinity for AUR. Notably, P^350^, L^351^, and L^378^ are nearly invariant. I^344^ is another largely conserved residue, with minor substitutions mainly by L and M. The basis for such high conservation of these residues is unclear.

## 4 Discussion

There is a long list of incurable diseases and multi-resistance microbes that are of global concern (Franco-Serrano et al., 2018; Vasan et al., 2019; Hoffman, 2020). Therefore, there is a pressing need to exploit new molecular targets to diversify treatments for these conditions. The malfunction of F_O_F_1_-ATP synthase is implicated in the development of multiple human diseases (Huang et al., 2008; Ahmad et al., 2013; Nesci et al., 2019; Patel et al., 2020). However, despite multiple efforts, no pharmacological modulator of this promising target has yet been approved for clinical treatment of noninfectious diseases. In contrast, with the authorization of bedaquiline as an antitubercular drug, this enzyme was validated as a species-specific antimicrobial target (Nesci et al., 2019). The idea of using exogenous inhibitors of F_O_F_1_-ATP synthase to treat human diseases is not new. Encouraging results have been reported to arrest the progression of different tumor cell lines through the inhibition of this enzyme by polyphenols, aurovertins, and glycomacrolides (Duan et al., 2020; Wu et al., 2020; Reisman et al., 2022). In principle, exogenous inhibitor binding sites could also be targeted to treat other diseases in which F_O_F_1_-ATP synthase is involved, although further research is still needed on this.

To our knowledge, this is the first study aimed at characterizing one of the non-functional F_O_F_1_-ATP synthase allosteric pockets to explore its druggability. Our results showed that the two AUR binding sites share similar conformational properties and recognition patterns, although an overall examination of the binding free energy, solvent site and hydrogen bonding results suggests that the β_TP_ site corresponds to the experimentally reported high-affinity site (Issartel et al., 1983a; Issartel and Vignais, 1984). Analysis of the conformational dynamics indicated that both AUR binding sites are preformed, in agreement with the relative invariance of these sites observed in all the experimental structures of BtF_1_ solved so far. Free energy calculations and solvent sites identification, two computational approaches, identified the same triad (R^412^, Y^458^, and Q^411^) as the most stabilizing residues for interaction with the inhibitor, results that are consistent with experimental site-directed mutagenesis data and with the different AUR sensitivities that enzymes of various species have (Lee et al., 1989). These residues could therefore be used as primary hot spots in drug design campaigns. Natural aurovertins differ from each other in their pyrone, bicyclo and/or linker substituents (Hong and Pedersen, 2008; Patel et al., 2020). Unfortunately, not enough experimental binding data have been reported so far to establish a quantitative structure-activity relationship for these inhibitors. Our simulations revealed that, in the catalytic dwell conformation, there is a clear interaction trend between β_TP_ and α_TP_ CTD residues that had not been reported before. This tendency, partially interrupted by AUR, seems to be in the formation pathway of a network of hydrogen bonds established between α_DP_ and β_DP_ that, according to mutational studies (Mao et al., 2008), is relevant for the correct functioning of the enzyme. Besides acting as a steric blocker of the propagation of protein conformational changes, AUR exerts long-range effects, increasing the conformational flexibility of the side chains and the nucleotide in the active site of the same catalytic subunit. These observations are consistent with experimental cooperative affinity effects between the inhibitor and the substrates (Issartel et al., 1983a; Issartel and Vignais, 1984).

Experimental characterization of AUR binding to F_1_ or entire F_O_F_1_-ATP synthase has been challenging, and numerous aspects of the inhibition mechanism remain to be unveiled Although the crystal structure revealed two molecules of AUR B bound to one F_1_, determination in solution of the stoichiometry (Verschoor et al., 1977; Issartel et al., 1983b; Johnson et al., 2009), binding affinities (Hong and Pedersen, 2008; Patel et al., 2020), and cooperative effects with the catalytic sites (Chang and Penefsky, 1973; Verschoor et al., 1977; Issartel and Vignais, 1984) has been challenging, with reported data varying depending on the conditions and measurement techniques, and which can be well described using different inhibition models (Johnson et al., 2009). In this sense, new experiments could be conceived based on the computational results presented here. For instance, mutations of key residues would be useful in improving our understanding of the mechanism of inhibition.

Free energy results for the interaction between AUR and F_1_ from two bacterial species shed new light on the possibilities of using the AUR binding site as an antimicrobial target. Although the overall identity between the BtF_1_ and EcF_1_ AUR binding sites is ∼80%, the eight energetically relevant residues for inhibitor binding are completely conserved. This conservation is consistent with the similar affinity values measured both *in silico* and *in vitro* for the two species. This suggests that the AUR binding site in EcF_1_ might not be a good target for the development of species-specific antibiotics. By extension, a similar conclusion can be suggested for *Coxiella*, *Haemophilus*, *Legionella*, and *Rickettsia* pathogens which cause Q fever, respiratory track, legionellosis, and Rocky Mountain spotted fever diseases, respectively. In contrast, the identity of the eight most important binding residues between BtF_1_ and MsF_1_ is 50%, which, according to the simulations, weakens the affinity and modifies the binding pose of AUR B. Indeed, these results are not unexpected, as mitochondria are accepted to have originated from an α-protobacteria-like ancestor (Roger et al., 2017; Fan et al., 2020), and *E. coli* is a γ-protobacteria. *M. smegmatis* is a more distantly related actinobacteria. Although it is unknown whether AUR inhibits MsF_1_, this difference in composition could be useful for the development of species-specific antimicrobial drugs to treat tuberculosis and other communicable diseases such as brucellosis (*Brucella sp*), botulism (*Clostridium botulinum*), cholera (*Vibrio cholera*), and diphtheria (*Corynebacterium diphtheriae*). An analysis of the AUR binding site of all bacterial sequences reported so far suggests that a significant number of species are insensitive to this class of inhibitor, and that varying degrees of affinity strength are likely to exist. Overall, our study lays the groundwork for structure-based drug design targeting the AUR binding site to treat human diseases in which F_O_F_1_-ATP synthase is centrally involved, or for the development of new types of antibiotics.

## Data Availability

The original contributions presented in the study are included in the article/Supplementary Materials, further inquiries can be directed to the corresponding author.
