# Supplementary material for: Exploring the druggability of the binding site of aurovertin, an exogenous allosteric inhibitor of FOF1-ATP synthase
Source: Front Pharmacol. 2022 Oct 14;13:1012008. doi: 10.3389/fphar.2022.1012008 (PMC9615146; doi:10.3389/fphar.2022.1012008)
Supplement: Supplementary file 1 [file DataSheet1.pdf]

Supplementary Material

Exploring the druggability of the binding site of aurovertin, an exogenous allosteric inhibitor of F_OF_1-ATP synthase

Luis Fernando Cofas-Vargas[1†], Paola Mendoza-Espinosa[1,2†], Luis Pablo Avila-Barrientos[1], Diego Prada-Gracia[3], Héctor Riveros-Rosas[4], and Enrique García-Hernández[1*]

†These authors contributed equally to this work and share first authorship

[1] Universidad Nacional Autónoma de México, Instituto de Química, Ciudad Universitaria, Ciudad de México, 04510 México.
[2] Tecnologico de Monterrey, The Institute for Obesity Research, Monterrey, Nuevo León 64849, Mexico.
[3] Unidad de Investigación en Biología Computacional y Diseño de Fármacos, Hospital Infantil de México Federico Gómez, Ciudad de México, 06720, México.
[4] Departamento de Bioquímica, Facultad de Medicina, Universidad Nacional Autónoma de México, Avenida Universidad 3000, Cd. Universitaria, Ciudad de México 04510, México.

*** Correspondence:**
Enrique García-Hernández,
egarciah@unam.mx

Table of content

**Table S1**	Atom types and partial charges used for AUR B
**Figure S1**	Conformational relaxation of the AUR binding site sites with and without inhibitor.
**Figure S2**	Contact analysis at the AUR binding site in β_E
**Figure S3**	Protein-inhibitor hydrogen bonding at the AUR binding site in β_E
**Figure S4**	Dihedral angle free energy landscapes (FEL) for the AUR binding site residues in β_E
**Figure S5**	Dihedral angle free energy landscapes (FEL) for α_{TP} residues
**Figure S6**	Cumulative per-residue squared covariance (σ^2) for the β_E site from a side chain dPCA
**Figure S7**	Conformational variability in the β_{TP} AUR binding site of experimental structures of BtF_1
**Figure S8**	Side chain dihedral angle free energy landscapes (FEL) for the nucleotide binding residues in β_E
**Figure S9**	Residue-wise free energy decomposition and solvent site identification in β_E

Table S1. Atom types and partial charges used for aurovertin B. The van der Waals parameters and bonded terms are those associated with these particular atom types in the Amber forcefield.

Atom name	Atom type	Partial charge	Atom name	Atom type	Partial charge
**C1**	c3	-0.0921	**H243**	hc	0.0790
**C2**	c3	-0.0904	**H242**	hc	0.0790
**C3**	c3	0.1101	**H241**	hc	0.0790
**C4**	c3	0.1328	**H233**	h1	0.0540
**C5**	c3	0.0851	**H232**	h1	0.0540
**C6**	c3	0.1448	**H231**	h1	0.0540
**C7**	c3	0.1051	**H223**	hc	0.0507
**C8**	c3	0.1263	**H222**	hc	0.0507
**C20**	c3	-0.0781	**H221**	hc	0.0507
**C21**	c3	-0.0841	**H213**	hc	0.0577
**C24**	c3	-0.1501	**H212**	hc	0.0577
**C25**	c	0.6381	**H211**	hc	0.0577
**O3**	os	-0.4006	**H203**	hc	0.0554
**O4**	os	-0.4026	**H202**	hc	0.0554
**O5**	os	-0.4259	**H201**	hc	0.0554
**O7**	oh	-0.5758	**H13A**	hc	0.0357
**O25**	o	-0.5380	**H12A**	hc	0.0357
**C9**	c2	-0.1422	**H11A**	hc	0.0357
**C10**	ce	-0.1520	**HO7**	ho	0.4030
**C11**	ce	-0.0960	**H9**	ha	0.1480
**C12**	cf	-0.1480	**H8**	h1	0.0777
**C13**	cf	-0.0580	**H7**	h1	0.0527
**C14**	ce	-0.1630	**H5**	h1	0.1067
**C15**	cc	0.1911	**H3**	h1	0.0687
**C16**	cd	-0.1982	**H22**	hc	0.0622
**C17**	cd	0.2351	**H21**	hc	0.0622
**C18**	cc	-0.3862	**H18**	ha	0.1710
**C19**	c	0.7398	**H14**	ha	0.1440
**C22**	c3	-0.0369	**H13**	ha	0.1520
**C23**	c3	0.1087	**H12**	ha	0.1280
**O15**	os	-0.3372	**H11**	ha	0.1340
**O17**	os	-0.3129	**H10**	ha	0.1290
**O19**	o	-0.5855			

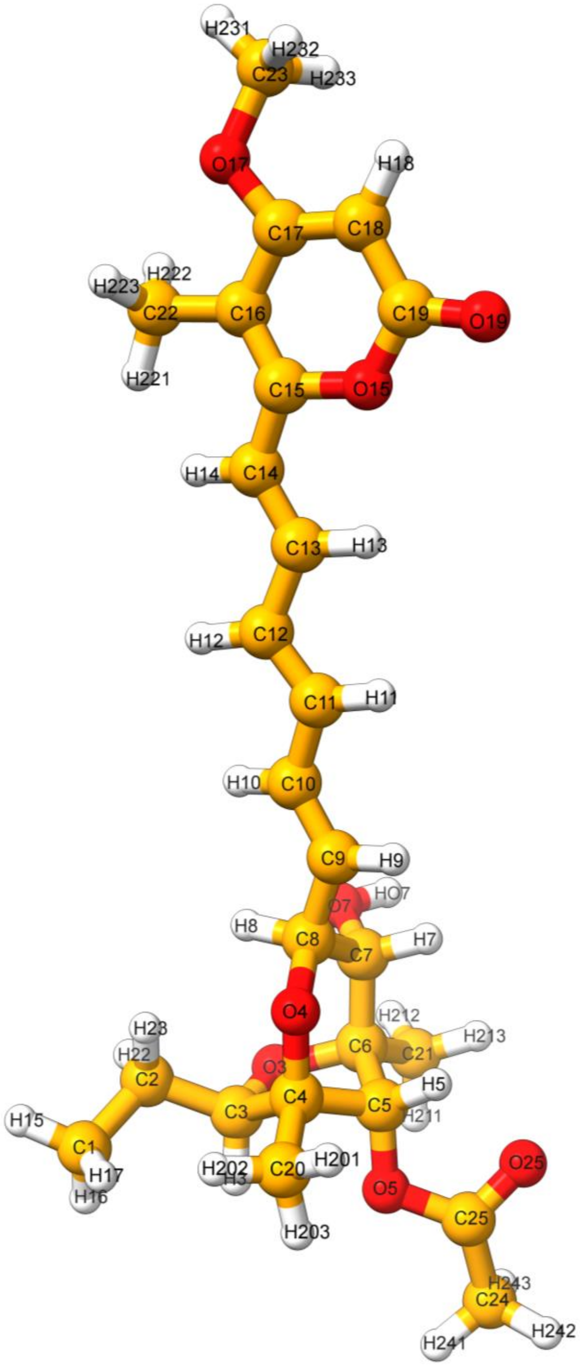

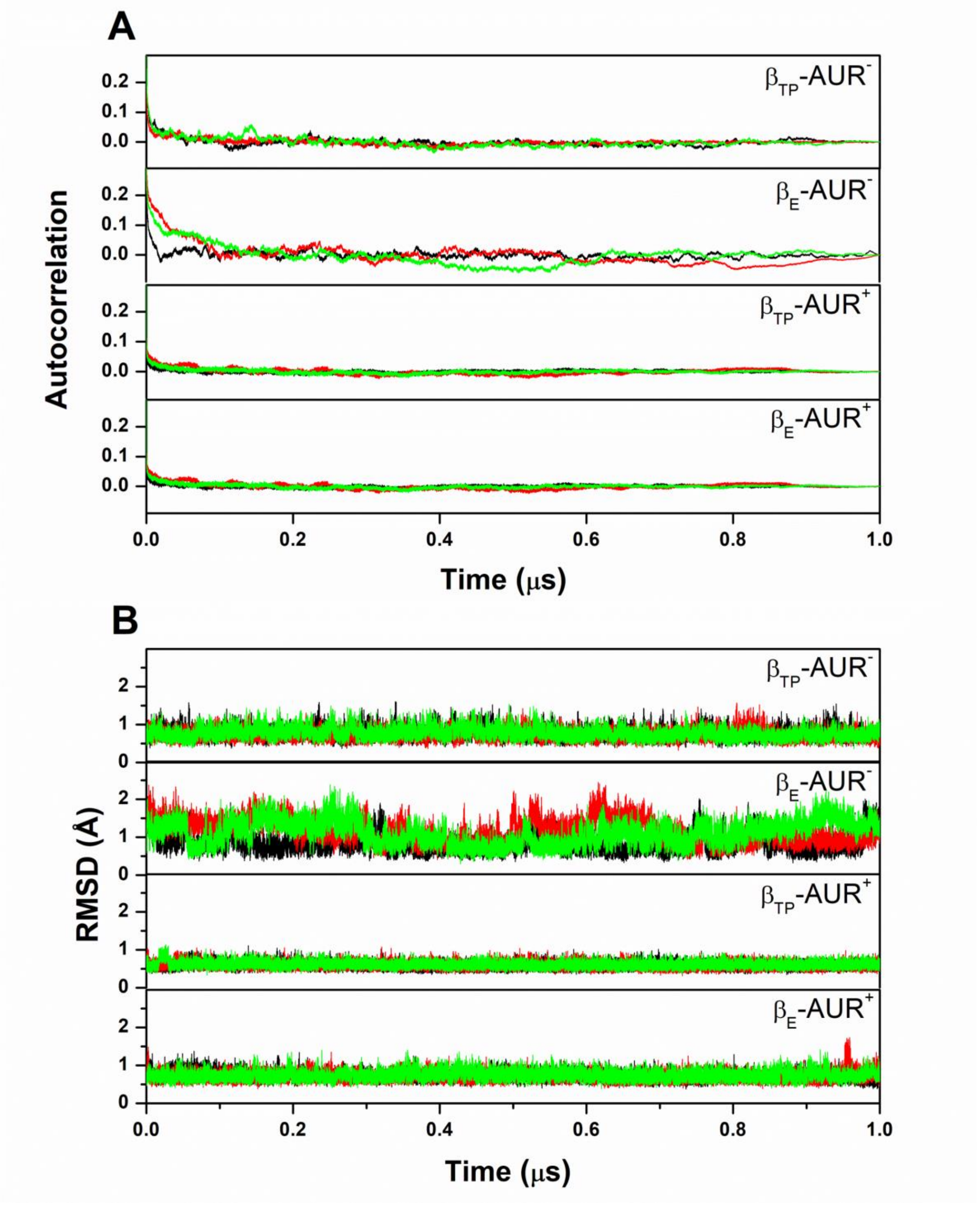

Figure S1. Conformational relaxation of the AUR binding sites with and without inhibitor. A). Autocorrelation function of backbone dihedral angles for AUR binding residues. For the autocorrelation analysis with CPPTRAJ, the square root of the sum of the squares of the values ϕ and ψ for each frame as a function of time was used. **B)** RMSD (over backbone heavy atoms) as a function of time. The crystal structure was used as reference. The three MD replicas are shown.

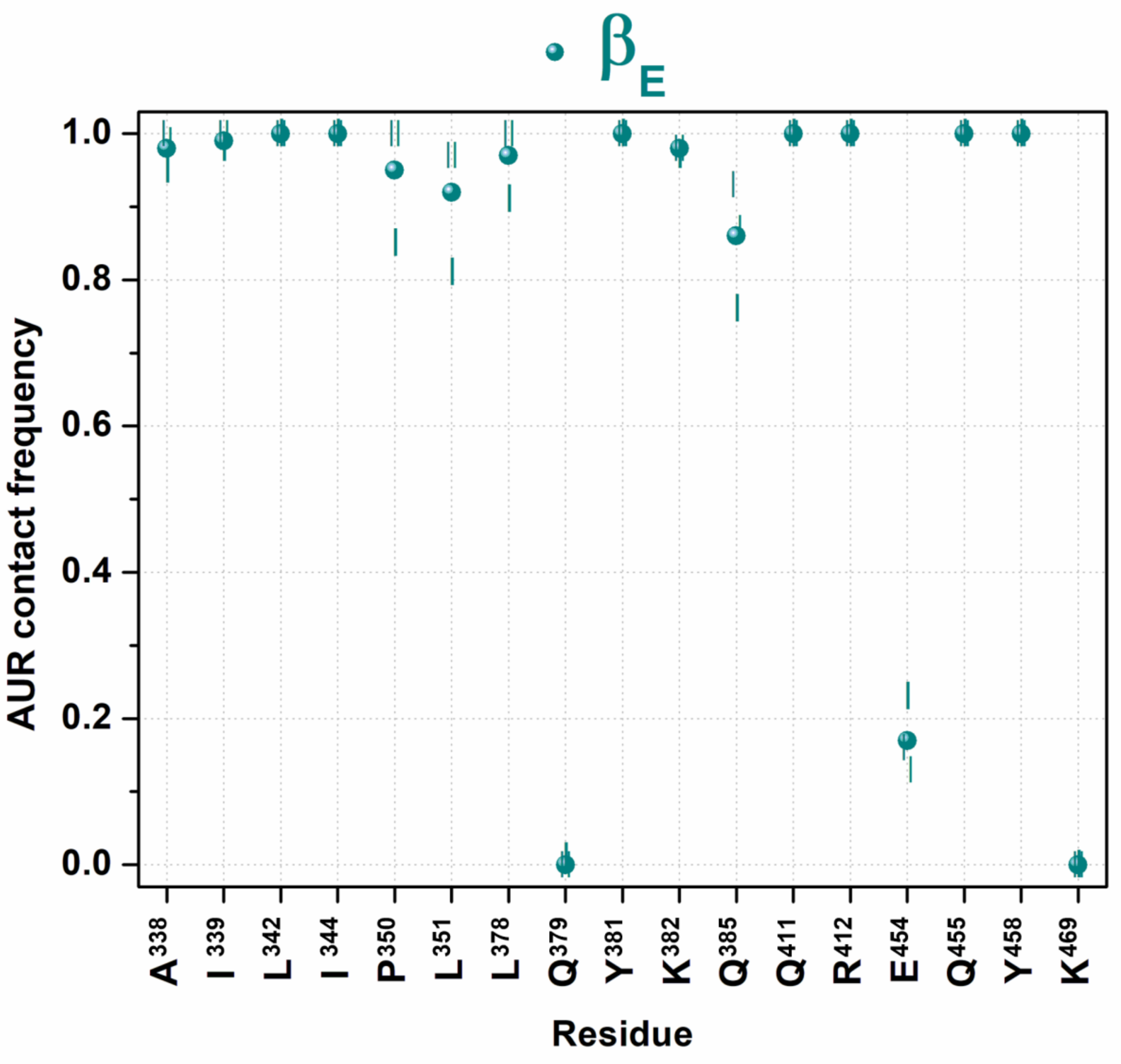

Figure S2. Contact analysis at the AUR binding site in β_E. AUR-protein interaction cumulative frequency observed in the MD simulations. Results for individual trajectories are shown as vertical lines; circle symbols correspond to the average values of the three replicas.

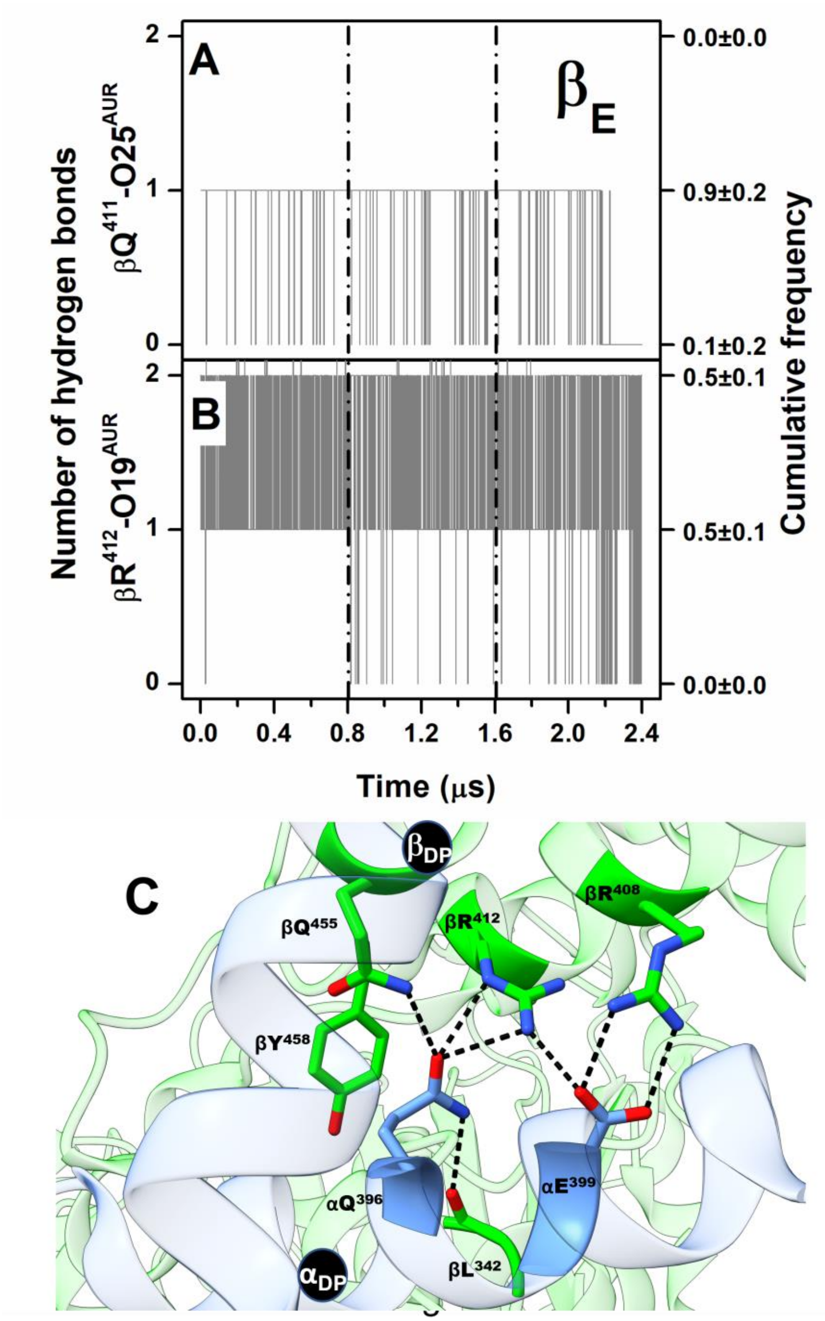

Figure S3. Protein-inhibitor hydrogen bonding at the AUR binding site in β_E and hydrogen bond network between CTD residues of α_{DP} and β_{DP}. A) βQ^{411}─O25. **B)** βR^{412}─O19. Data for the three concatenated replicas are shown, after subtracting the first 0.2 μs of simulation from each of them. The trajectories for each replica are delimited by dashed lines. Cumulative frequencies refer to the total fraction of time that each number of hydrogen bonds was observed in the simulations. **C)** Hydrogen bonds formed by α_{DP} (in blue) and β_{DP} (in green) residues, occluding the AUR binding site.

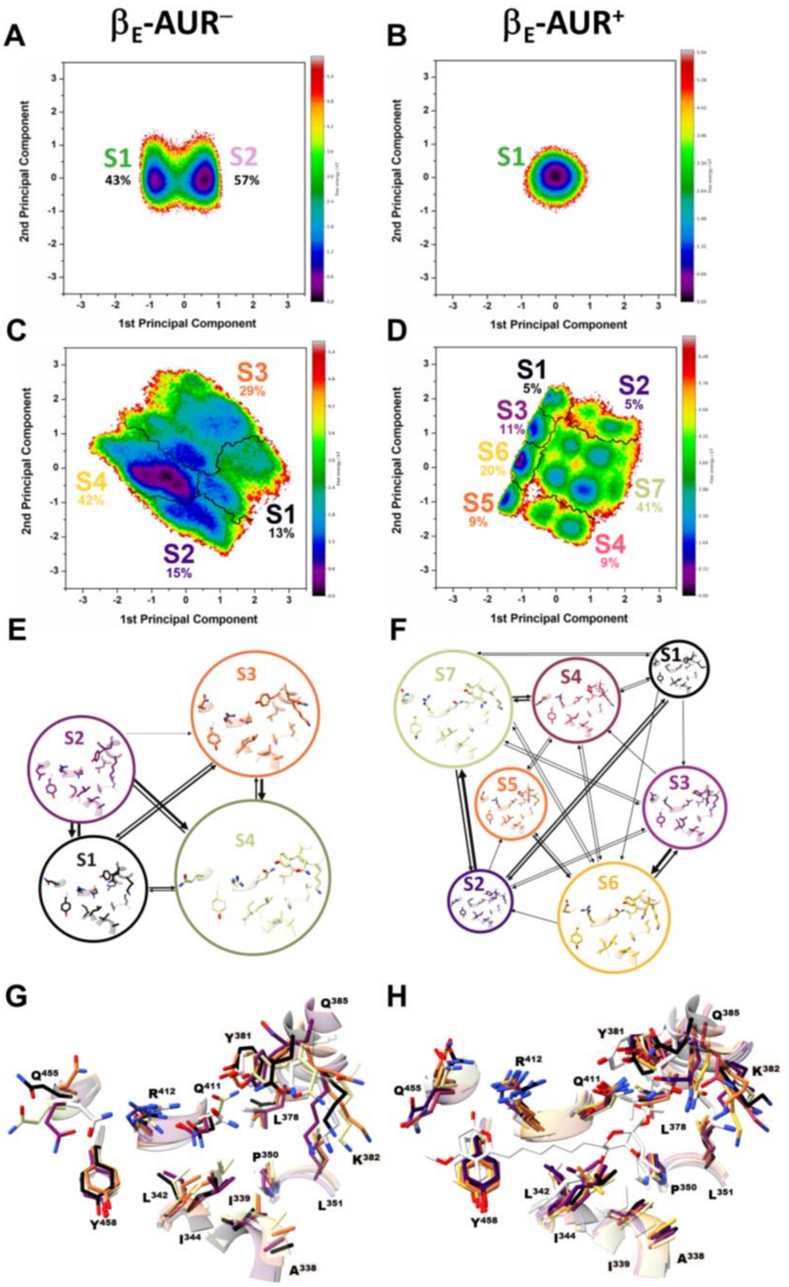

Figure S4. Dihedral angle free energy landscapes (FEL) for the AUR binding site residues in β_E. FEL (in k_BT units) were obtained from a dPCA projected onto the first two principal components in the absence (left) and presence (right) of the inhibitor. **A,B)** Backbone dPCA. One and two metastable conformational states were observed for AUR^+ (S1) and AUR^- (S1,S2), respectively. The percentage of cumulative frequencies are shown. The main difference between S1 and S2 was the ψ angle value of I^{344}. **C,D)** Side chain dPCA. Black lines delimit the macrostates identified through a Markov-state model analysis. **E,F**) Network transition pathway of the Markov-state model. The thickness of the connecting arrows is proportional to the transition probability. **G,H)** Superimposition of r**e**presentative conformations for each attraction basin in **E,F)**. Macrostates were labeled S1, S2 and so on from lowest to highest occupancy.

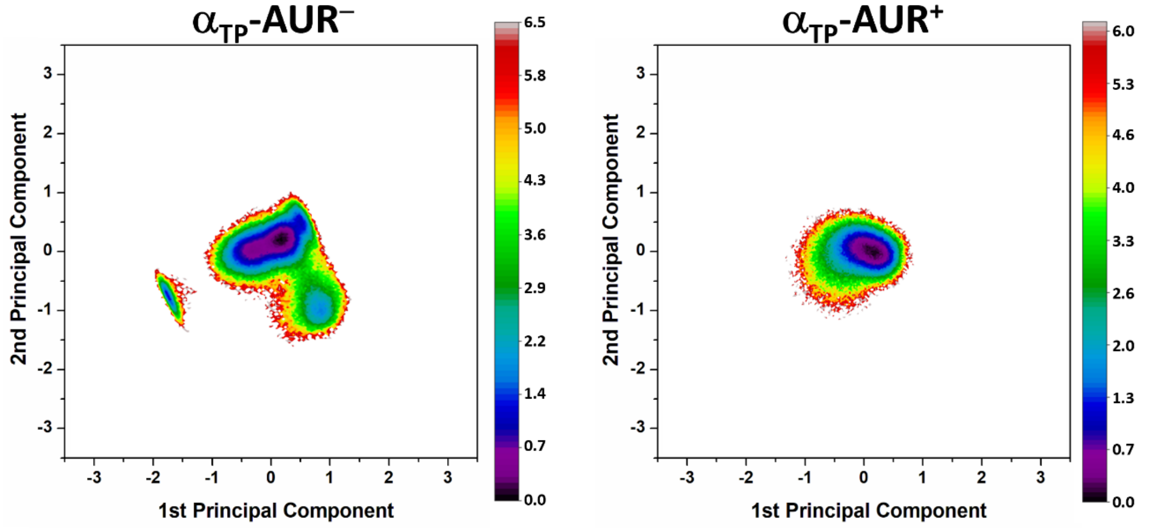

Figure S5. Dihedral angle free energy landscapes (FEL) for α_{TP} residues. L^{392}, E^{393}, A^{395}, Q^{396}, and E^{399} contacted AUR in the β_{TP} binding site. FEL (in k_BT units) were obtained from a backbone dPCA projected onto the first two principal components in the absence (left) and presence (right) of the inhibitor.

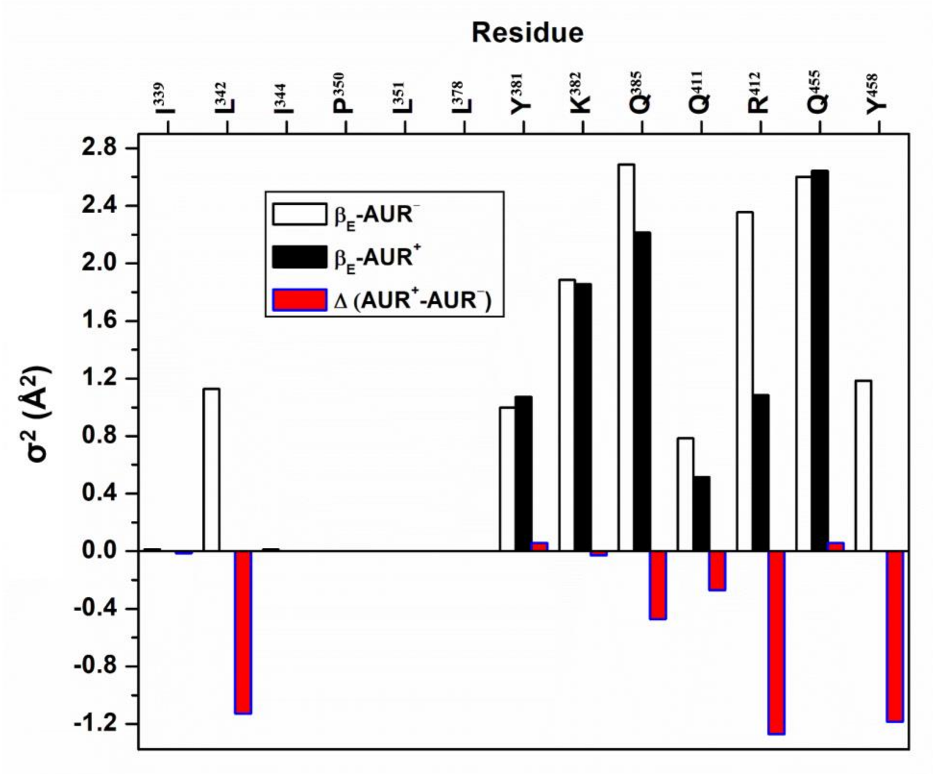

Figure S6. Cumulative variance per residue (σ^2) for the β_E site from a side chain dPCA. $\Delta(AUR^+ - AUR^-)$ is the σ^2 difference in presence minus in absence of the inhibitor. Values correspond to 70% of the total variance.

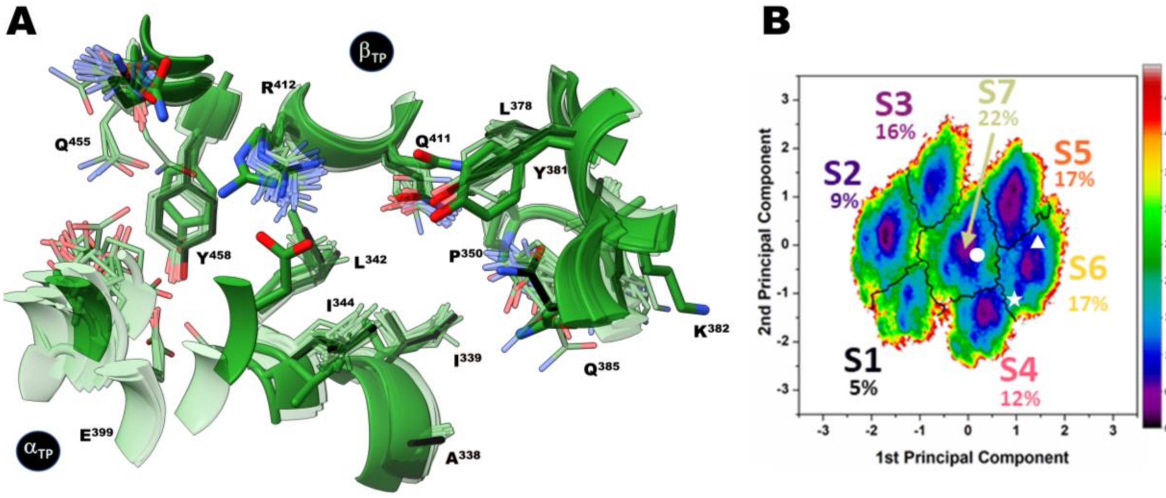

Figure S7. Conformational variability in the β_{TP} AUR binding site in experimental BtF_1 structures. **A)** A search for BtF_1 structures deposited in the PDB at better than 3.5 Å resolution returned a total of 28 structures: **one in complex with aurovertin** (PDB ID: 1cow (van Raaij et al., 1996); **seven in complex with IF1** (PBD IDs: 2v7q (Gledhill et al., 2007b), 1ohh (Cabezón et al., 2003), 4tsf, 4tt3 (Bason et al., 2014), 4z1m (Bason et al., 2015) and 6zqm ((Spikes et al., 2020); **one in complex with azide** (PDB ID 2ck3 (Bowler et al., 2006); **three in complex with polyphenols** (PDB IDs: 2jiz, 2jj1, 2jj2 (Gledhill et al., 2007a)); **one in complex with efrapeptin** (PDB ID: 1efr (Abrahams et al., 1996); **one in complex with DCCD** (PDB ID:1e79 (Gibbons et al., 2000)); **two in complex with AlF_4** (PDB IDs: 1h8e (Menz et al., 2001b), and 1e1r (Braig et al., 2000)); **one in complex with BeF_4** (PDB ID: 1w0j (Kagawa et al., 2004)); **one apo structure** (PDB ID: 2w6j (Sanchez-Weatherby et al., 2009); **two dimer structures** (PDB IDs: 6zpo and 6zqn (Spikes et al., 2020); **10 with variable nucleotide binding site occupancies** (PDB IDs: 1e1q (Braig et al., 2000), 1h8h (Menz et al., 2001a), 1nbm (Orriss et al., 1998), 1w0k, (Kagawa et al., 2004), 4asu (Rees et al., 2012), 2wss (Rees et al., 2009), 4yxw (Bason et al., 2015)), 6ziu (Spikes et al., 2020), 6yy0 (Spikes et al., 2020), 1bmf (Abrahams et al., 1994)), and **one ground state structure** (PDB ID 2jdi (Bowler et al., 2007)). F_1 structures were superimposed on the solved BtF_1 structure in complex with AUR B (side chains shown in black sticks). All other crystal structures are in semi-transparent wireframe. The most visited MD conformer is shown in green sticks. **B)** Side chain dihedral angles of the crystal structures were calculated and the corresponding coordinates were projected onto FEL of βTP-AUR⁻. The side chain conformations in most crystal structures (white circle) fell within S7 macrostate. The conformations observed in 6yy0 and 1cow (white triangle and star, respectively) corresponded to S6 macrostate.

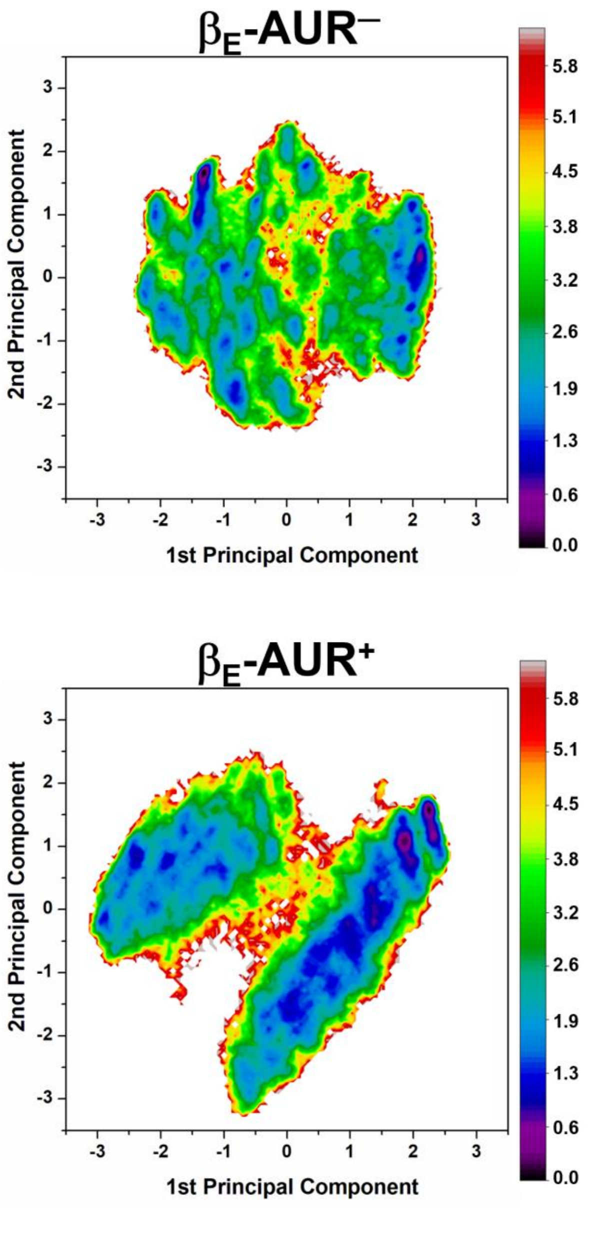

Figure S8. Side chain dihedral angle free energy landscapes (FEL) for nucleotide binding residues in β_E. FEL (in k_BT units) were obtained from a dPCA projected onto the first two principal components.

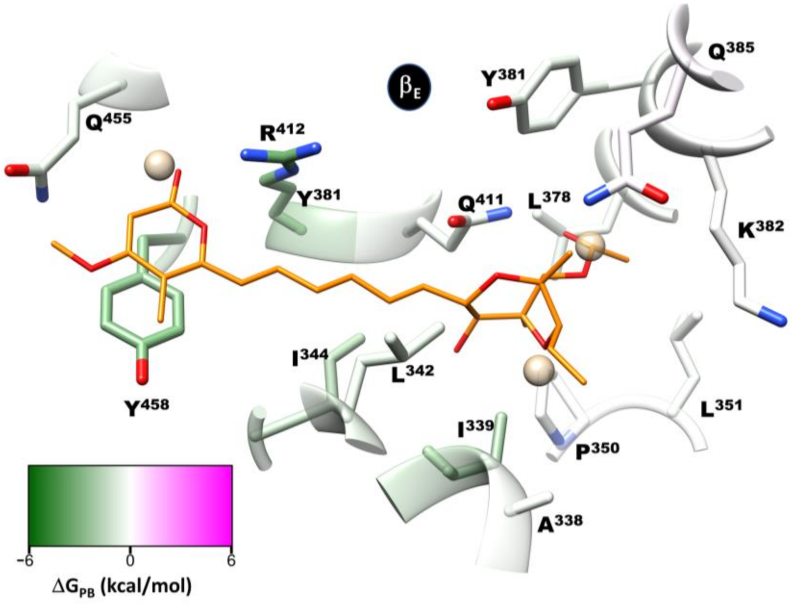

Figure S9. Per-residue free energy decomposition and solvent site identification in β_E. Per-residue decomposition of the binding free energy (ΔG_{PB}) was calculated with the MMPBSA method. Residues that favor interaction with the inhibitor are shown in green. The identified hydrophobic solvent sites (SS_{HP}) are shown as spheres.

References

Abrahams, J. P., Buchanan, S. K., Van Raaij, M. J., Fearnley, I. M., Leslie, A. G. W., and Walker, J. E. (1996). The structure of bovine F1-ATPase complexed with the peptide antibiotic efrapeptin. *Proc. Natl. Acad. Sci. U. S. A.* 93, 9420–9424. doi: 10.1073/pnas.93.18.9420.

Abrahams, J. P., Leslie, A. G. W., Lutter, R., and Walker, J. E. (1994). Structure at 2.8 Â resolution of F1-ATPase from bovine heart mitochondria. *Nature* 370, 621–628. doi: 10.1038/370621a0.

Bason, J. V, Montgomery, M. G., Leslie, A. G. W., and Walker, J. E. (2014). Pathway of binding of the intrinsically disordered mitochondrial inhibitor protein to F 1 -ATPase. *Proc. Natl. Acad. Sci.* 111, 11305–11310. doi: 10.1073/pnas.1411560111.

Bason, J. V, Montgomery, M. G., Leslie, A. G. W., and Walker, J. E. (2015). How release of phosphate from mammalian F 1 -ATPase generates a rotary substep. *Proc. Natl. Acad. Sci.* 112, 6009–6014. doi: 10.1073/pnas.1506465112.

Bowler, M. W., Montgomery, M. G., Leslie, A. G. W., and Walker, J. E. (2006). How azide inhibits ATP hydrolysis by the F-ATPases. *Proc. Natl. Acad. Sci.* 103, 8646–8649. doi: 10.1073/pnas.0602915103.

Bowler, M. W., Montgomery, M. G., Leslie, A. G. W., and Walker, J. E. (2007). Ground state structure of F1-ATPase from bovine heart mitochondria at 1.9 Å resolution. *J. Biol. Chem.* 282, 14238–14242. doi: 10.1074/jbc.M700203200.

Braig, K., Menz, R. I., Montgomery, M. G., Leslie, A. G., and Walker, J. E. (2000). Structure of bovine mitochondrial F1-ATPase inhibited by Mg2+ADP and aluminium fluoride. *Structure*. doi: 10.1016/S0969-2126(00)00145-3.

Cabezón, E., Montgomery, M. G., Leslie, A. G. W., and Walker, J. E. (2003). The structure of bovine F1-ATPase in complex with its regulatory protein IF1. *Nat. Struct. Biol.* 10, 744–750. doi: 10.1038/nsb966.

Gibbons, C., Montgomery, M. G., Leslie, A. G., and Walker, J. E. (2000). The structure of the central stalk in bovine F(1)-ATPase at 2.4 A resolution. *Nat. Struct. Biol.* 7, 1055–61. doi: 10.1038/80981.

Gledhill, J. R., Montgomery, M. G., Leslie, A. G. W. W., and Walker, J. E. (2007a). Mechanism of inhibition of bovine F1-ATPase by resveratrol and related polyphenols. *Proc. Natl. Acad. Sci.* 104, 13632–13637. doi: 10.1073/pnas.0706290104.

Gledhill, J. R., Montgomery, M. G., Leslie, A. G. W., and Walker, J. E. (2007b). How the regulatory protein, IF 1 , inhibits F 1 -ATPase from bovine mitochondria. *Proc. Natl. Acad. Sci.* 104, 15671–15676. doi: 10.1073/pnas.0707326104.

Kagawa, R., Montgomery, M. G., Braig, K., Leslie, A. G. W., and Walker, J. E. (2004). The structure of bovine F1-ATPase inhibited by ADP and beryllium fluoride. *EMBO J.* 23, 2734–2744. doi: 10.1038/sj.emboj.7600293.

Menz, R. I., Leslie, A. G. W., and Walker, J. E. (2001a). The structure and nucleotide

occupancy of bovine mitochondrial F1-ATPase are not influenced by crystallisation at high concentrations of nucleotide. *FEBS Lett.* 494, 11–14. doi: 10.1016/S0014-5793(01)02302-X.

Menz, R. I., Walker, J. E., and Leslie, A. G. W. (2001b). Structure of bovine mitochondrial F1-ATPase with nucleotide bound to all three catalytic sites: Implications for the mechanism of rotary catalysis. *Cell* 106, 331–341. doi: 10.1016/S0092-8674(01)00452-4.

Orriss, G. L., Leslie, A. G., Braig, K., and Walker, J. E. (1998). Bovine F1-ATPase covalently inhibited with 4-chloro-7-nitrobenzofurazan: the structure provides further support for a rotary catalytic mechanism. *Structure* 6, 831–837. doi: 10.1016/S0969-2126(98)00085-9.

Rees, D. M., Leslie, A. G. W., and Walker, J. E. (2009). The structure of the membrane extrinsic region of bovine ATP synthase. *Proc. Natl. Acad. Sci. U. S. A.* 106, 21597–601. doi: 10.1073/pnas.0910365106.

Rees, D. M., Montgomery, M. G., Leslie, A. G. W., and Walker, J. E. (2012). Structural evidence of a new catalytic intermediate in the pathway of ATP hydrolysis by F1-ATPase from bovine heart mitochondria. *Proc. Natl. Acad. Sci.* 109, 11139–11143. doi: 10.1073/pnas.1207587109.

Sanchez-Weatherby, J., Bowler, M. W., Huet, J., Gobbo, A., Felisaz, F., Lavault, B., et al. (2009). Improving diffraction by humidity control: a novel device compatible with X-ray beamlines. *Acta Crystallogr. Sect. D Biol. Crystallogr.* 65, 1237–1246. doi: 10.1107/S0907444909037822.

Spikes, T. E., Montgomery, M. G., and Walker, J. E. (2020). Structure of the dimeric ATP synthase from bovine mitochondria. *Proc. Natl. Acad. Sci. U. S. A.* 117, 23519–23526. doi: 10.1073/pnas.2013998117.

van Raaij, M. J., Abrahams, J. P., Leslie, A. G. W. W., and Walker, J. E. (1996). The structure of bovine F1-ATPase complexed with the antibiotic inhibitor aurovertin B. *Proc. Natl. Acad. Sci. U. S. A.* 93, 6913–6917. doi: 10.1073/pnas.93.14.6913.
